# Inorganic High-Performance Fiber-Based Materials for Electromagnetic Interference Shielding: Fundamentals, Fabrications, and Emerging Applications

**DOI:** 10.1007/s40820-025-02053-z

**Published:** 2026-01-30

**Authors:** Sijie Qiao, Zhicheng Shi, Aixin Tong, Zhiyu Huang, Annan He, Binhao Wang, Jun He, Jiaxin Wang, Ming Chen, Zixi Huang, Linhui Hao, Bing Wu, Yan Jun, Ya-Lan Tan, Pibo Ma, Weilin Xu, Fengxiang Chen

**Affiliations:** 1 State Key Laboratory of New Textile Materials and Advanced Processing, Wuhan Textile University, Wuhan, 430200 People’s Republic of China; 2 China Coal Technology & Engineering Group (CCTEG), Chinese Institute of Coal Science (CICS), Beijing, 100013 People’s Republic of China; 3 Engineering Research Center of Knitting Technology, Ministry of Education, College of Textile Science and Engineering, Jiangnan University, Wuxi, 214122 People’s Republic of China

**Keywords:** Inorganic high-performance fibers/fabrics, Electromagnetic shielding, Mechanism, Atomic layer deposition, Electromagnetic protection, Future challenges

## Abstract

Confronted with increasingly severe challenges of electromagnetic interference (EMI) and electromagnetic radiation pollution in industrial, military, and aerospace applications, the development of novel materials that combine high shielding efficiency with excellent comprehensive performance has become a research hotspot. Inorganic high-performance fibers (IHPFs), recognized for their lightweight nature, outstanding mechanical properties, and chemical stability, are regarded as ideal candidate materials for designing lightweight, durable, and structurally functional integrated EM shielding systems. However, besides metal fibers, most IHPFs exhibit intrinsic surface chemical inertness and physical smoothness, resulting in poor interfacial compatibility and weak adhesion with functional coatings or resin matrices, which significantly undermine the long-term service reliability of composites under extreme conditions. This paper introduces the EM shielding mechanism, highlights common issues of surface inertness in IHPFs, and elaborates on both “dry” and “wet” surface modification strategies. These strategies enable the formation of robust functional layers, facilitating the integration of high strength, high modulus, and multifunctionality, while ensuring interfacial reliability in composites. Furthermore, the principles and processing techniques of various strategies for fabricating EMI shielding functional layers on IHPFs surfaces are reviewed, and recent advances in the application of functionalized IHPFs, as well as service reliability and environmental stability, are summarized, including EMI shielding protection and radar-absorbing stealth. Finally, the challenges and future research directions for the large-scale and long-term stable application of IHPF-based EMI shielding functionalization in high-end fields are discussed, offering insights that may accelerate the development of next-generation lightweight, sustainable, and multifunctional EMI shielding materials.

## Highlights

- Inorganic high-performance fibers (IHPFs)-based composites development and electromagnetic interference (EMI) shielding mechanisms are reviewed.
- Surface modification strategies for IHPF’s surface inertness challenge and EMI shielding layer construction are summarized.
- Future directions and current challenges for achieving large-scale, durable, and environmentally stable IHPF-based EMI shielding materials are outlined.

## Graphical abstract

**Figure:**
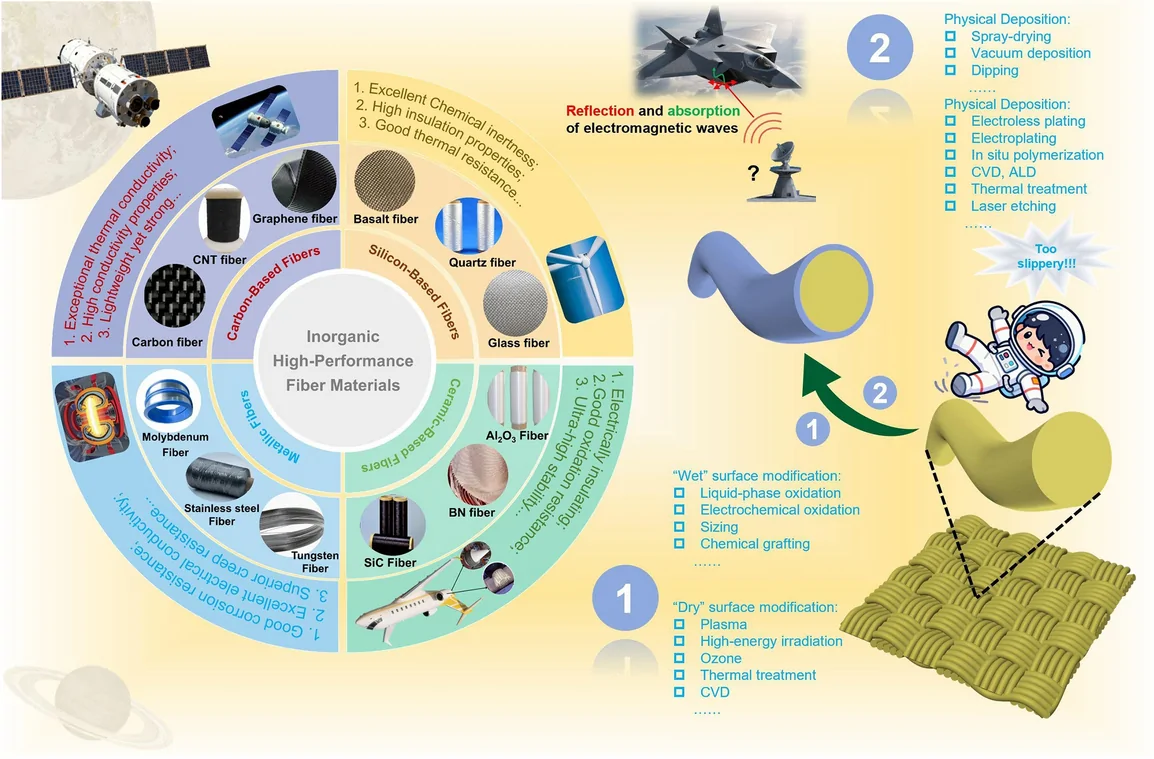

## Introduction

With the rapid development of wireless communication, radar technology, and the widespread use of gigahertz power devices, the space we live in has become saturated with dense EM waves. The resulting electromagnetic interference (EMI) and electromagnetic radiation (EMR) pollution have emerged as significant issues that cannot be ignored. They not only lead to malfunctions of precision electronic devices and jeopardize information security, but also pose potential threats to human health [1–3]. In extreme environments such as aerospace, additional natural factors including strong ultraviolet radiation and cosmic rays further affect the orderly operation of space stations and the health of astronauts. Traditional metallic shielding materials (e.g., copper and aluminum foils), though exhibiting excellent electrical conductivity and shielding performance, suffer from inherent drawbacks such as high density, susceptibility to corrosion, rigidity, and poor processability. These limitations severely restrict their application in modern electronic devices that demand lightweight, flexible, and highly integrated materials [4, 5]. Consequently, it is urgent to develop high-efficiency, lightweight, and durable EMI shielding materials suitable for aerospace, military, and other extreme conditions.

**Fig. 1:**
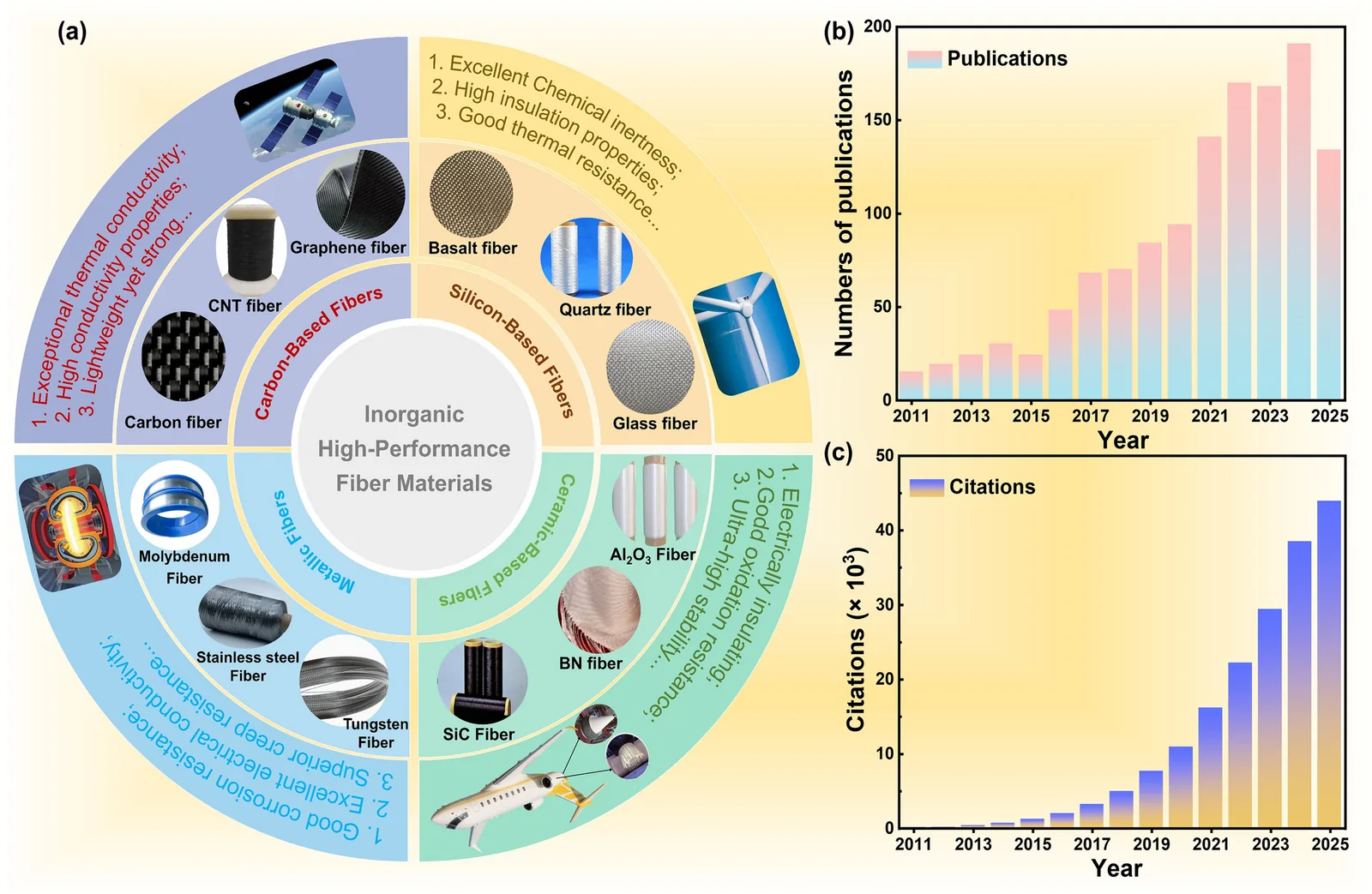
**a** Inorganic high-performance fiber materials used in EMI shielding with their corresponding functional characteristics. **b, c** Numbers of publications and citations regarding IHPF materials with EMI shielding properties from 2011 to 2025. The data were collected on the Web of Science with the key words of “Carbon fiber”, “Graphene fiber”, “CNT fiber”, “Basalt fiber”, “Quartz fiber”, “Glass fiber”, “SiC fiber”, “BN fiber”, “Al_2_O_3_ fiber”, “Molybdenum fiber”, “Tungsten fiber”, “Stainless steel fiber”, and “EMI Shielding”

Among the candidate materials, inorganic high-performance fibers (IHPFs) have attracted growing attention due to their unique structural and excellent comprehensive properties. Generally, IHPFs can be broadly classified into traditional IHPFs and emerging nanomaterial-based fibers. The traditional IHPFs include glass fibers (GFs), quartz fibers (QFs), basalt fibers (BFs), SiC fibers, and other ceramic fibers. Their high strength-to-weight ratios, excellent thermal stability, and remarkable chemical inertness make them ideal structural materials for aerospace and defense applications (Fig. 1 and Table 1) [6–8]. In contrast, the emerging nanomaterial-based fibers—such as carbon nanotube (CNT) fibers, graphene fibers, MXene fibers, and hybrid nanofibers—exhibit outstanding electrical conductivity, flexibility, and multifunctionality derived from their nanoscale building blocks [9–11]. However, except for metallic and carbon-based fibers [12, 13], most traditional IHPFs are insulating in nature, making it difficult to realize effective reflection and absorption of EM waves [14–16]. Moreover, these fibers share inherent challenges: their highly stable chemical structures and smooth surfaces result in low surface energy and a scarcity of reactive sites, which can easily lead to delamination of functional coatings under stress or thermal cycling, ultimately causing the loss of EMI shielding functionality. This has become a critical bottleneck affecting their functional modification and the overall performance of fiber-reinforced composites [17–19].

**Table 1:** Mechanical and physical properties and applications of IHPFs

Fiber category	Fiber types	Key characteristics	Main applications	References
Carbon-based fiber	CF	Ultra-high specific strength; Good fatigue resistance; Excellent electrical conductivity	Aircraft & spacecraft structures, sporting goods	[54]
	Graphene fiber	Lightweight yet strong; Superior flexibility; Outstanding EMI shielding ability	Wearable sensors, flexible supercapacitors, batteries	[100]
	CNT fiber	Outstanding specific strength; Low density; Ultra-high electrical conductivity	EMI shielding wires, flexible conductors	[99]
Silicon-based fiber	BF	Excellent thermal stability; Good flame retardancy; Environmentally friendly	Fireproof fabrics, sporting goods, wind blades	[95]
	GF	High tensile strength at low cost; Chemical inertness; Easy processability	Antenna covers, boat hulls, wind blades	[91]
	QF	Outstanding thermal shock resistance; Electrically insulating	High-frequency radomes, precision optical components	[92]
Ceramic-based fiber	SiC fiber	Outstanding oxidation resistance; corrosion-proof; Superior radiation resistance	Rocket nozzles, jet-engine hot-section parts (blades, vanes)	[105]
	Si_3_N_4_ fiber	Chemically inert; Outstanding neutron & radiation tolerance	Hypersonic vehicle radomes	[104]
	Al_2_O_3_ fiber	Ultra-high temperature stability; Low thermal conductivity; Chemical inertness	Furnace linings, metal-matrix brake pads, lightweight armor	[102]
	BN fiber	Low density & lightweight; Good flexibility & weaveability	Rocket combustion-chamber liners, antenna radomes	[103]
Metal fiber	Molybdenum fiber	Superior creep resistance; Good corrosion resistance Ideal for extreme-environment	High-temperature furnace elements, hypersonic thermal protection systems	[12]
	Tungsten fiber	Highest melting point; Low thermal expansion coefficient; Excellent electrical conductivity	Rocket nozzle reinforcements, radiation shields	[13]

To overcome these intrinsic limitations of traditional IHPFs, surface activation and interfacial modification have become indispensable strategies. Addressing the surface inertness of IHPFs not only enables the construction of robust functional layers on their surfaces—integrating high strength, high modulus, and multifunctionality—but also promotes strong interfacial adhesion with various polymer matrices, ensuring excellent composite performance. Therefore, necessary surface activation pretreatments, aimed at increasing surface roughness and introducing reactive chemical groups, serve as the prerequisite and key initial step for building durable EMI shielding coatings. Considerable research has been devoted to improving fiber surface activity. Currently, the most widely explored approaches include liquid-phase oxidation [20, 21], electrochemical oxidation [22, 23], plasma treatment [24, 25], surface sizing [26, 27], coating [28, 29], and chemical grafting [30, 31]. Although electrochemical oxidation has been extensively applied in industry, harsh modification conditions may impair fiber mechanical properties. Plasma treatment offers high efficiency, but entails high costs and potential surface damage. Surface sizing and coating methods, which apply a compatible polymer layer onto fiber surfaces, can effectively protect the fibers and are relatively simple, though they may encounter interfacial bonding issues with polymer matrices. Surface grafting, on the other hand, has emerged as an important strategy for fiber functionalization, offering strong design flexibility, controllable and mild reaction conditions, and minimal damage to fiber mechanical properties. However, challenges remain regarding the control of grafting degree and uniformity. Each method presents distinct advantages and disadvantages, requiring careful selection based on practical application needs.

After successful surface activation, precise functionalization strategies become critical for imparting EMI shielding and absorption capabilities to the fibers. The central objective of these strategies lies in constructing continuous conductive networks and incorporating magnetic or dielectric loss components. Conventional physical deposition and chemical treatment methods, as well as emerging interfacial modification techniques, represent the primary pathways. Physical deposition techniques such as magnetron sputtering [32–34], vacuum-assisted filtration [35, 36], and spraying [37, 38] can rapidly form metallic or carbon-based thin films on fiber surfaces, thereby enhancing conductivity and shielding performance, though challenges of adhesion and durability remain. In contrast, electroless plating [39–41], electroplating [42, 43], and chemical vapor deposition (CVD) [44–46] can produce dense, continuous metallic or carbon-based coatings with superior electrical conductivity and wear resistance. By tailoring parameters such as coating thickness, grain size, and interfacial morphology, the shielding mechanism—including reflection, absorption, and multiple scattering—can be finely regulated. Additionally, in situ polymerization [47–49] and surface grafting [50, 51] can introduce conductive polymers (e.g., polyaniline, polypyrrole) onto fiber surfaces, yielding lightweight, flexible composites that balance mechanical integrity with EM loss performance. In recent years, with advances in nanotechnology, novel strategies such as atomic layer deposition (ALD) [52–54] and laser etching [55, 56] have been increasingly applied to the functional modification of IHPFs. ALD, with its atomic-level precision in layer-by-layer deposition, enables the formation of uniform, ultra-thin, and strongly adherent conformal coatings, providing new opportunities for interfacial engineering and multifunctional integration. Laser etching, by directly creating micro–nanostructures on fiber surfaces, enhances interfacial roughness and increases multiple scattering pathways of EM waves, thereby significantly improving absorption performance. Taken together, the continuous evolution of surface functionalization strategies is offering greater design flexibility and broader application prospects for IHPF-based EMI shielding materials.

Although significant progress has been achieved in the study of IHPFs for EMI shielding, there is still a lack of systematic reviews summarizing their preparation methods, application scenarios, and future challenges. This review aims to fill that gap by providing a comprehensive overview of this rapidly evolving field. First, the mechanisms of EMI shielding and corresponding evaluation criteria are discussed. Then, the inherent challenges of IHPFs are analyzed, along with current approaches to overcome them and their respective advantages and limitations. Subsequently, we focus on the functionalization strategies for imparting EMI shielding properties to IHPFs, covering both traditional physical and chemical methods as well as emerging interfacial modification techniques. Furthermore, their innovative applications in extreme environments such as aerospace, advanced electronics, and stealth defense are elaborated. Finally, the major challenges and future directions in this field are outlined, aiming to provide valuable insights and inspiration for the design of the next generation of structurally integrated, multifunctional EMI shielding materials.

## Fundamental Principles for Electromagnetic Shielding

### Electromagnetic Shielding Mechanisms

Shielding refers to the suppression of EMI by “cutting off” the coupling paths of EM fields, and it is one of the primaries means of achieving protection against EM radiation. EM shielding involves the use of conductive or magnetic materials to confine EMR within a specified spatial region. The purpose is either to enclose the interference source with a shielding body to suppress its disturbance to sensitive equipment or to protect personnel in the surrounding space, or to enclose sensitive equipment to prevent interference from external sources. In general, EMI shielding refers to shielding against alternating EM fields above 10 kHz [57–60]. According to EM theory, in high-frequency EM waves characterized primarily by radiation, the electric and magnetic fields are interdependent. Therefore, in practice, shielding either the electric or the magnetic field is sufficient, since the other will be eliminated simultaneously.

**Fig. 2:**
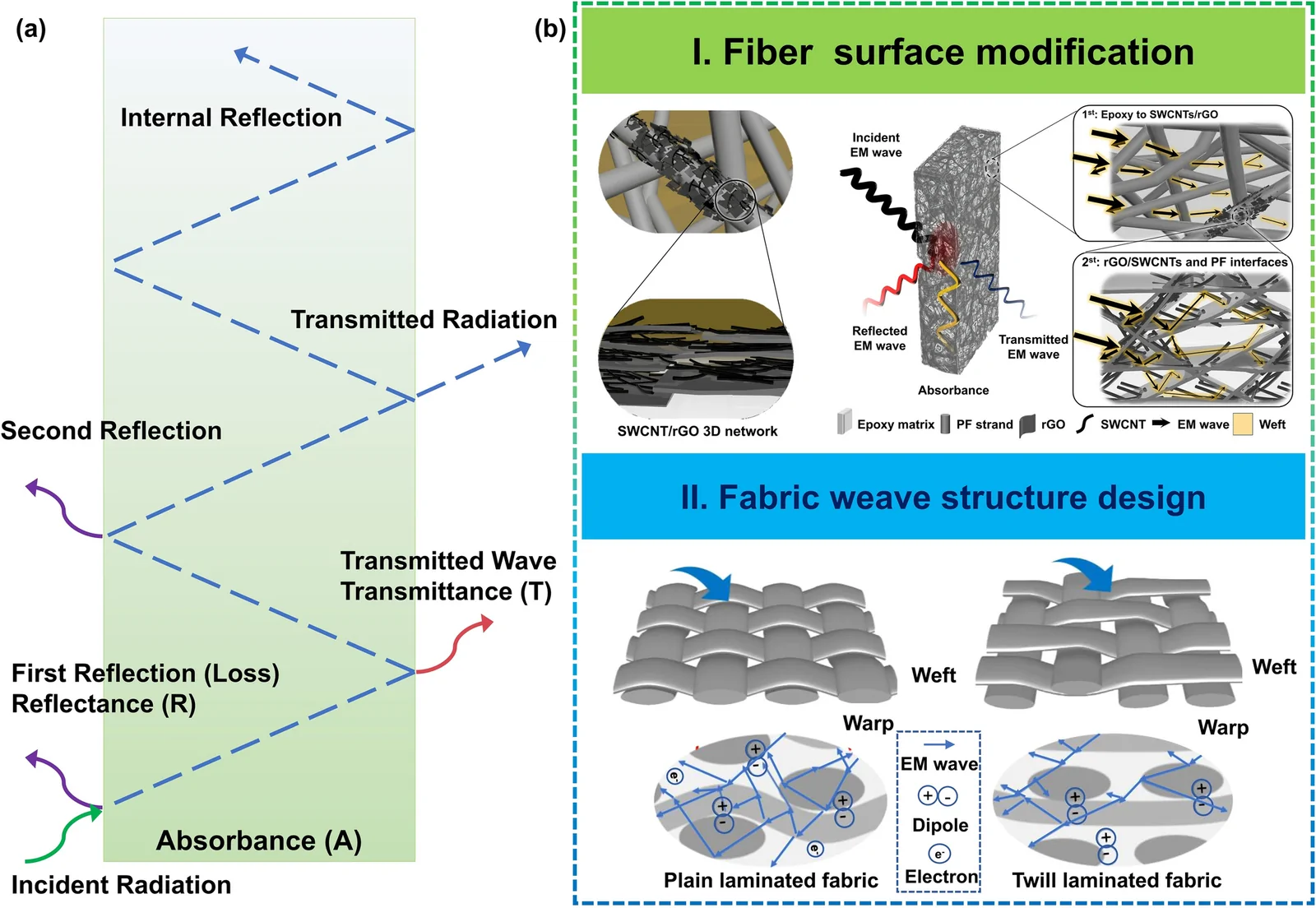
Electromagnetic shielding mechanism. **a** Transmission line theory. **b** From fiber surface modification to fabric weave structure design. **I.** The EMI shielding mechanisms for the carbon ink-loaded PF/epoxy composites; **II.** Fabric weave structure design: EMI shielding mechanism of plain-weave structure and twill weave structure. Reproduced with permission from [85]. Copyright 2021, Elsevier B.V

The mechanisms of EMI shielding can be explained using various approaches, such as the eddy current effect method [61, 62], EM field theory, and transmission line theory. Among these, the transmission line theory has become widely adopted because of its computational simplicity, high accuracy, and intuitive understanding. As illustrated in Fig. 2a, transmission line theory treats the shielding body as a segment of a transmission line. When a radiation field encounters the shielding material, part of it is reflected at the outer surface, while the remainder penetrates into the shield and propagates forward [63–65]. During propagation, the EM waves undergo continuous attenuation within the shield, along with multiple reflections and transmissions at its interfaces. Therefore, the shielding mechanism comprises three components: reflection loss (SE_R_) on the surface of the shield, absorption loss (SE_A_) within the shielding material, and multiple reflection loss (SE_M_) inside the shield.

Based on Schelkunoff’s theory [66], the total EM shielding effectiveness (SE_T_) can be expressed as the combined contribution of SE_R_, SE_A_, and SE_M_ for the EM waves transmitted through the shielding body, as shown in Eq. (1):

1 $${\mathrm{SE}}_{{\text{T }}} {\text{ = SE}}_{{\mathrm{R}}} {\text{ + SE}}_{{\mathrm{A}}} {\text{ + SE}}_{{\mathrm{M}}}$$

The intensity of EM waves penetrating into a conductor decreases progressively with increasing depth. The skin depth (δ) is defined as the distance at which the EM waves intensity decreases to 1/*e* (*e* is the Euler’s number; 1/*e* = 0.37) [67–69]. The expression for δ is given as:

2 $$\delta = \frac{1}{\alpha } = \left( {\sqrt {\pi f\sigma \mu } } \right)^{ - 1}$$

Here, *σ* denotes the electrical conductivity, *f* the radiation frequency, and *μ* the magnetic permeability. The parameter α is the attenuation constant of EM waves in the shielding material, expressed as $\alpha = \omega \sqrt {\frac{\mu \varepsilon }{2}\left[ {\sqrt {1 + \left( {\frac{\sigma }{\omega \varepsilon }} \right)^{2} - 1} } \right]}$, where *ω* represents the angular frequency (2π*f*) and *ε* the dielectric constant. From Eq. (2), δ decreases with increasing *f*, *σ*, and *μ*.

#### SE_*R*_

The shielding effectiveness due to reflection arises from the impedance mismatch between the propagation medium of the incident EM waves and the surface of the shielding material. When an incident EM wave encounters a conductive material with high carrier density, a portion of the wave is reflected due to the discontinuity in the intrinsic impedance. Therefore, SE_R_ is primarily determined by the electrical conductivity and carrier mobility of the shielding material. The SE_R_ value from the front to the back surface of the shielding layer can be expressed as Eq. (3):

3 $$SE_{R } = 20\log_{10} \left( {\frac{{Z + Z_{0} }}{{4ZZ_{0} }}} \right) = 39.5 + 10\log_{10} \frac{\sigma }{2f\pi \mu }$$

Here, *Z* and *Z*_*0*_ represent the impedances of the shielding material and air, respectively. According to Eq. (3), SE_R_ is related to the material’s σ and μ as well as the frequency *f* of the EM waves [70].

#### SE_*A*_

SE_A_ represents the attenuation of the EM waves energy as it propagates through the material. The incident wave induces eddy currents and dipole polarization losses within the shielding layer, which convert EM energy into heat [71]. To significantly enhance absorption loss, the following conditions are required: (i) high electrical conductivity for Ohmic loss, which strengthens the interaction between electrons and incident EM waves; and (ii) high *σ* and *μ*, which increase eddy current loss and hysteresis loss. The absorbed EM energy is dissipated in the form of heat. The SE_A_ of the shielding material can be expressed as Eq. (4):

4 $$SE_{A} = 20\left( {\frac{d}{\delta }} \right)\log_{10} e = 8.68\frac{d}{\delta } = 8.68d\sqrt {f\mu \sigma }$$

where *d* denotes the thickness of the shielding layer. Thus, SE_A_ depends on controllable parameters such as thickness and conductivity, as well as the intrinsic EM properties of the material.

#### SE_*M*_

SE_M_ accounts for the energy dissipation caused by multiple internal reflections at interfaces or within porous/multilayer structure This process can repeat until the EM energy is fully dissipated. The multiple reflection efficiency (SE_M_) of a shielding material is calculated as Eq. (5):

5 $$SE_{M} = 20\log_{10} \left( {1 - e^{{\frac{ - 2d}{\delta }}} } \right) = 20\log_{10} \left( {1 - 10^{{\frac{{SE_{A} }}{10}}} } \right)$$

SE_M_ mainly depends on the thickness of the material. When the thickness of the shielding layer exceeds the skin depth or when SE_T_ > 15 dB, SE_M_ can be neglected [72, 73]. However, if the thickness is significantly smaller than the skin depth, multiple reflections must be considered when evaluating the shielding performance.

In addition to macroscopic reflections occurring between the two boundaries of the EMI shielding layer, reflections and scattering can also take place within the microstructure of the shielding material. Such internal multiple reflections and scattering can further extend the propagation path of EM waves, thereby enhancing absorption loss and overall SE.

In summary, SE_R_ is dominant in highly conductive metallic coatings, SE_A_ is crucial for magnetic or dielectric loss materials, and SE_M_ becomes significant only in thin or low-loss multilayer systems where internal reflections cannot be fully attenuated. Understanding the interplay among these terms provides valuable guidance for designing materials with balanced reflection–absorption behavior in EMI shielding applications. It is worth noting that in certain cases, excessive reflection of EM waves may deteriorate the EM environment in the surrounding space. Therefore, researchers have continuously focused on increasing the absorption loss of EMI shielding materials, reducing their reflection loss, and mitigating their environmental impact [74–77].

### Evaluation Criteria

To quantitatively describe the shielding performance, SE is commonly used to evaluate the ability and efficiency of a shielding material in suppressing EMI. SE is influenced by the properties of the shielding material, the frequency of the interference source, the distance between the shielding body and the interference source, as well as various possible discontinuities present in the shield [78, 79]. The SE can be expressed as shown in Eq. (6):

6 $$SE = 20\log_{10} \left( {\frac{{E_{t} }}{{E_{i} }}} \right) = 20\log_{10} \left( {\frac{{H_{t} }}{{H_{i} }}} \right) = 20\log_{10} \left( {\frac{{P_{t} }}{{P_{i} }}} \right)$$

Here, *E*_*i*_, *E*_*t*_, *H*_*i*_, *H*_*t*_, *P*_*i*_, and *P*_*t*_ represent the incident electric field strength, transmitted electric field strength, incident magnetic field strength, transmitted magnetic field strength, incident power, and transmitted power, respectively.

In experiments, EMI SE is typically determined using a vector network analyzer (VNA) by measuring the scattering parameters S_11_ and S_21_. S_11_ represents the portion of the EM wave emitted from port 1 that is reflected by the shielding material and received again at port 1, while S_21_ represents the portion of the EM wave emitted from port 1 that passes through the shielding material and is received at port 2. Based on Eqs. (7–12), the R, T, A, SE_T_, SE_R_, and SE_A_ can be calculated.

7 $${\text{R = }}\left| {{\mathrm{S}}_{{{11}}} } \right|^{{2}}$$

8 $${\text{T = }}\left| {{\mathrm{S}}_{{{21}}} } \right|^{{2}}$$

9 $${\text{A = 1}} - {\mathrm{R}} - {\mathrm{T}}$$

10 $${\mathrm{SE}}_{T} = - 10\log (T)$$

11 $${\mathrm{SE}}_{{\mathrm{R}}} { = } - {\mathrm{10log}}\left( {{1} - {\mathrm{R}}} \right){ = } - {\mathrm{10log}}\left( {{1} - \left| {{\mathrm{S}}_{{{11}}} } \right|^{{2}} } \right)$$

12 $${\mathrm{SE}}_{{\mathrm{A}}} { = } - {\mathrm{10log}}\left( {{\mathrm{T}}/{(1} - {\mathrm{R)}}} \right){ = } - {\mathrm{10log}}\left( {\left| {{\mathrm{S}}_{{{21}}} } \right|^{{2}} /{(1} - \left| {{\mathrm{S}}_{{{11}}} } \right|^{{2}} )} \right)$$

The functionalization of EMI shielding in fibers is usually achieved through two approaches. One is to deposit conductive layers and magnetic substances on fibers’ surface [80–84]. The other is to change the fabric’s weaving method from a structural design perspective, altering the transmission direction of EM waves on the fiber surface [85], thereby enhancing the effect of EMI shielding (Fig. 2b). Moreover, in the field of wearable devices, shielding materials are required not only to meet the demands of EMI shielding but also to be “thin” and “light”, thereby enhancing the comfort of wearable electronics. In aerospace, integrated circuits, and related fields, lightweight materials can effectively reduce overall weight, saving both energy and space. To evaluate material performance while fully accounting for the effects of thickness and density on SE, we define the following three specific SE parameters (SSE, SE/t, and SSE/t) [86–89]:

13 $${\text{SSE = }}\frac{{\text{EMI SE}}}{{\uprho }}\left( {{\text{dB cm}}^{{3}} {\text{ g}}^{{ - 1}} } \right)$$

14 $${\mathrm{SE}}/{\text{t = }}\frac{{\text{EMI SE}}}{{\mathrm{d}}}\left( {{\text{dB cm}}^{{ - 1}} } \right)$$

15 $${\mathrm{SSE}}/t = \frac{{{\mathrm{EMISE}}}}{{\rho \cdot d}}\left( {{\mathrm{dBcm}}^{2} {\mathrm{g}}^{{ - 1}} } \right)$$

The SSE incorporates three key parameters—SE, thickness (*d*), and density (*ρ*)—and is particularly important for evaluating the EMI SE of lightweight and thin materials. A higher SSE value indicates that the material is thinner and lighter while still maintaining strong shielding performance. Normalized parameters have been widely applied in the fields of porous EMI shielding materials and ultra-thin EMI shielding materials, especially for multilayer heterogeneous EMI shielding systems. Unlike single-layer materials, multilayer composite materials usually exhibit a synergistic effect due to interface impedance mismatch, multiple internal reflections, and distributed conductive or magnetic loss centers. Therefore, while the absolute SE value reflects the total attenuation capability, SSE provides a weight-normalized shielding efficiency metric, which is crucial for lightweight aerospace and wearable applications. Similarly, SE/t represents thickness-normalized shielding performance and is suitable for ultra-thin coatings and flexible fabrics. The comprehensive index SSE/t further quantifies the shielding per unit mass and thickness.

## Common Problem and Modification Strategies for High-Performance Inorganic Fiber Materials

Although IHPFs possess superior intrinsic properties, their application in EMI shielding is often hindered by interfacial challenges. Across different fiber systems, one recurring bottleneck emerges—surface inertness. This universal issue governs the extent to which functional coatings or matrix resins can adhere to the fiber surface, directly influencing the integrity and durability of the shielding network. A clear understanding of this common problem is therefore essential before exploring modification strategies to overcome it.

### Common Problem for IHPFs

IHPFs constitute a class of continuous filament materials manufactured via high-temperature melt-drawing or CVD processes from inorganic compounds or elements (e.g., SiO_2_, Al_2_O_3_, SiC, C). Owing to their lightweight nature, exceptional mechanical properties (high specific strength and modulus), outstanding thermal stability, and chemical inertness, they are regarded as ideal structural–functional integrated carriers. These fibers are extensively employed in cutting-edge fields such as aerospace, defense, and industrial infrastructure. Common types include GFs [90, 91], QFs [92], BFs [93–95], CFs [96–98], CNTs fibers [99], graphene fibers [100], and ceramic fibers (e.g., Al_2_O_3_, BN, SiC, Si_3_N_4_ fiber) [101–105]. However, their highly stable chemical structures and smooth physical morphologies result in intrinsic surface inertness, which presents common problems for their application in EMI shielding (Table 2). For instance, CFs exhibit a carbon content exceeding 90%, with carbon atoms arranged in a highly ordered structure, leading to pronounced surface inertness [106]. This inertness is primarily manifested as extremely low surface energy and a severe lack of reactive sites, resulting in poor interfacial compatibility and weak physical/chemical adhesion between the fiber surface and functional coatings (e.g., metals, conductive polymers, or carbon nanomaterials). The underlying cause lies in the fact that the intrinsic chemical bonds (e.g., C–C, Si–O, Al–O bonds) of most inorganic fibers (except metallic fibers) are highly stable and covalent, making effective chemical interactions with external substances difficult. This inherent “inertness” can have catastrophic consequences in composite systems; under external stress or thermal cycling, weak interfacial bonding readily becomes the initiation point for failure, causing functional coatings to powder, crack, or delaminate extensively from the fiber surface. Ultimately, this disrupts the EMI shielding network, leading to rapid performance degradation or complete failure. Therefore, effectively overcoming this common challenge through surface activation strategies to establish a robust and durable foundation for subsequent functional coatings is a prerequisite for realizing high-performance and highly reliable fiber-based EMI shielding materials. This section will systematically analyze the nature of this interfacial issue and provide a detailed review of advanced surface treatment strategies to address it.

**Table 2:** Advantages and disadvantages of IHPFs

Advantages	Disadvantages
Ultra-high temperature resistance	High manufacturing cost
High strength-to-weight ratio	Intrinsic brittleness (low strain-to-failure)
Excellent chemical & corrosion resistance	Difficult to cut or machine
Superior creep and fatigue resistance	Limited flexibility
Outstanding thermal stability & low CTE	Poor impact toughness
Electrically/thermally conductive or insulating as desired	Surface inertness → weak fiber-matrix bonding
Tailorable dielectric/EMI shielding properties	Requires specialized sizing or coupling agents
Non-flammable and radiation tolerant	Dense (not lightweight for some ceramic fibers)
Long service life under extreme environments	Limited supply chain & long lead times
Enables multifunctional composites	High processing temperatures (> 1000 °C) for sintering/CVI

### Modification Strategies

Addressing the surface inertness of IHPFs not only enables the construction of robust functional layers on the fiber surface, integrating high strength, high modulus, and multifunctionality, but also promotes strong interfaces with various resin matrices, ensuring superior composite performance. Consequently, necessary surface activation pretreatment to increase surface roughness and introduce active chemical groups is a critical prerequisite and the essential first step for constructing durable EMI shielding functional coatings. Based on the phase and medium environment in which the physicochemical processes occur at the fiber interface during treatment, various methods can be categorized into “dry” and “wet” processes (Table 3). It should be noted that the surface modification strategies discussed in this section primarily aim to activate the chemically inert surfaces of IHPFs and improve their interfacial reactivity and adhesion with subsequent coatings or matrices. These activation processes serve as fundamental pretreatments prior to EMI functionalization, while the strategies to construct the EMI shielding functional layers is systematically discussed later in Sect. 4.

**Table 3:** The surface treatment method (to address surface inertness) of IHPFs and the corresponding core mechanism, advantages, and disadvantages

Processing method		Core mechanism	Advantages	Disadvantages	Key parameters	References
“Dry” surface modification	Plasma treatment	Introducing polar groups and etching	High efficiency, environmentally friendly	Time-limited issue, high equipment cost	Flexural** s**trength (109.19 MPa)	[108]
	High-energy irradiation	Breaking surface’s molecular chains	High penetration power, no need for vacuum, batch processing	High equipment cost, radiation hazards	IFSS (47.23 → 68.57 MPa)	[110]
	Ozone	Oxidizing and introducing oxygen-containing functional groups	Simple operation, no liquid-phase contamination	Weak oxidation capacity, needing tail gas treatment	MW heating Performance 158 → 203 °C	[119]
	Thermal treatment	Surface lattice reconstruction or generate active surfaces	Simple process, increasing the degree of crystallization	Affecting fibers’ mechanical properties, poor controllability	N_2_, 1000 °C (tensile strength: 1.26 GPa)	[120]
	Chemical vapor deposition	Gaseous precursors form a solid coating through chemical reactions	High-purity coating, capable of complex shaping	High-temperature damage to fibers, high cost of precursors	1000–1200 °C (deposition); TiCl_4_ → Ti (H_2_)	[116]
“Wet” chemical modification	Liquid-phase oxidation	Strong acids or oxidizing agents erode fiber	Relatively simple, relatively high efficiency	Damage to fibers, heavy contamination	Oxidation time: 10–30 min	[21]
	Electrochemical oxidation	Forming oxygen-containing groups and micro-grooves	Uniform, controllable	High equipment cost	Tensile strength: 65.1 → 94.5 MPa	[23]
	Surface sizing	Coat fibers’ surface with a compatible polymer coating	Protect fibers, easy to operate	Limited temperature resistance	IFSS: 9.5 → 20.68 MPa	[27]
	Chemical grafting	Specific functional groups are grafted	Lasting effect, controllable reaction conditions	Grafting ratio, homogeneity	IFSS: 43.6 → 89.6 MPa	[31]

#### “Dry” Surface Modification

**Fig. 3:**
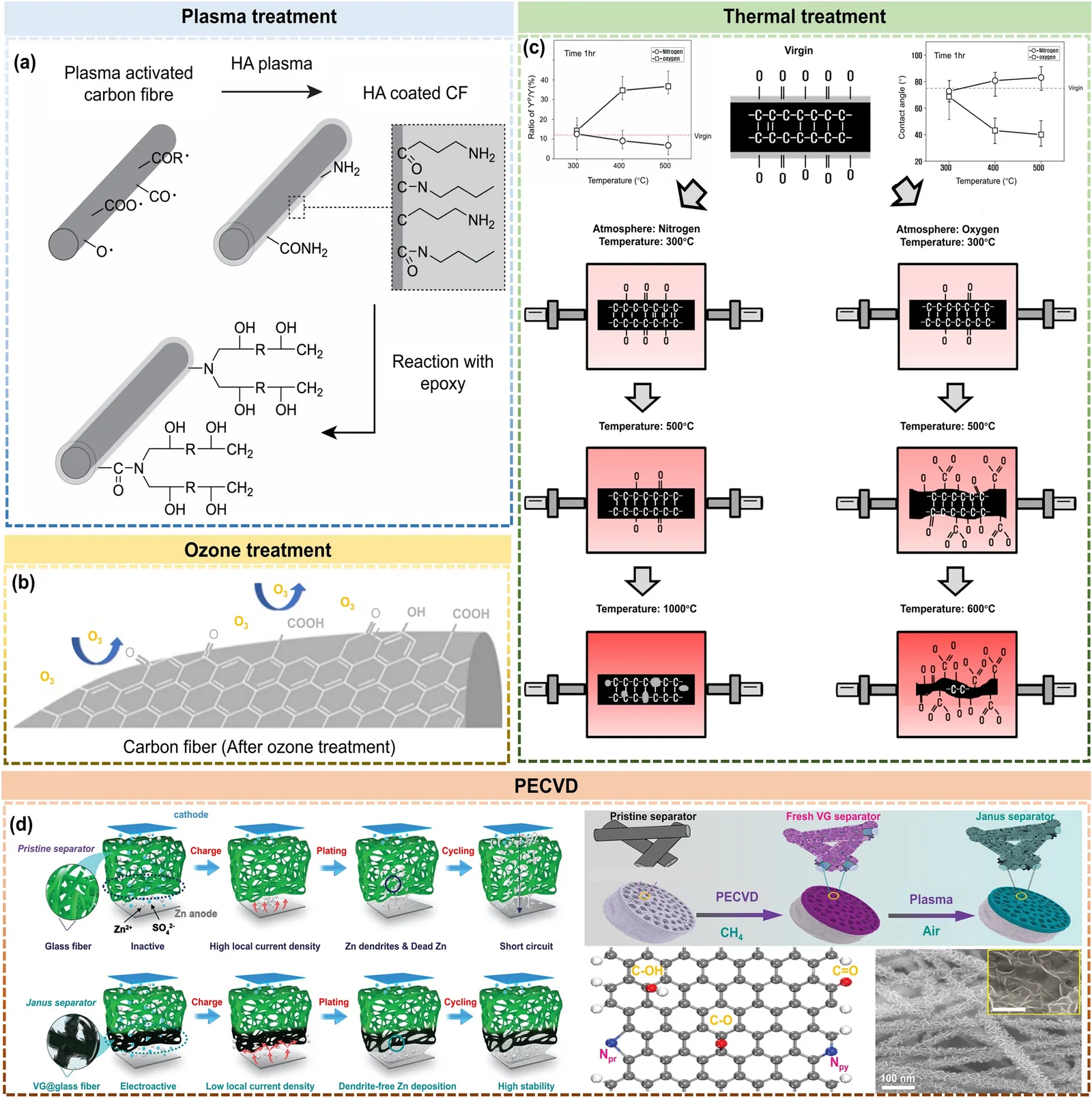
“Dry” surface modification method for the inert surface of IHPFs: plasma treatment method; ozone; thermal treatment method. **a** HA plasma treatment process and resulting interface with epoxy matrix; The relationship between IFSS, flexural strength (σ_*b*_), and plasma activation-treatment time. Reproduced with permission from [118]. Copyright 2024, Elsevier B.V.; **b** CF’s surface after ozone treatment. Reproduced with permission [119]. Copyright 2025, Elsevier B.V.; **c** Surface activity of CFs under different heat treatment environments and temperatures. Reproduced with permission [120]. Copyright 2022, MDPI;** d** Design of Janus separator targeting stabilized Zn anode; Synthetic process of Janus separator; Configuration of O and N-doped graphene. Reproduced with permission [121]. Copyright 2020, John Wiley & Sons

Dry processing refers to methods where the fiber surface does not directly contact liquid media (particularly aqueous solutions) during treatment. Reactions or interactions typically occur in gaseous or vacuum environments, such as plasma treatment [107–109], high-energy irradiation [110], ozone treatment [111–113], thermal treatment [114], and vapor deposition [115–117]. Plasma comprises electrons, ions, neutrals, radicals, excited atoms, molecules, and photons, all generated by electron-driven reactions. Plasma surface treatment is highly efficient; its core mechanism involves bombarding the fiber surface with high-energy active species (e.g., oxygen plasma) to introduce polar groups and cause etching. Yang et al. implemented a three-step process of Ar plasma cleaning, O_2_ plasma functionalization, and HA plasma polymerization, achieving a synergistic interfacial strengthening effect. After 3 min of treatment, the interfacial shear strength (IFSS) of CF/epoxy composites increased from 39.3 MPa to a maximum of 81.4 MPa (Fig. 3a) [118]. Ozone treatment is another typical dry surface treatment technology. Its core mechanism utilizes the strong oxidizing power of gaseous ozone (O_3_) to induce redox reactions on the fiber surface at room temperature, selectively introducing oxygen-containing functional groups and mildly etching the surface, thereby altering the fiber's surface chemical activity and physical structure. Huang et al. subjected pitch-based CFs to ozone treatment, successfully introducing stable oxygen-containing polar functional groups such as carboxyl groups (-COOH), resulting in CFs with enhanced microwave heating performance (Fig. 3b) [119]. Thermal treatment, as an important dry surface treatment technology, can effectively modify the surface chemistry of fibers by controlling the atmosphere and temperature, thereby improving their interfacial compatibility with resin matrices. The core mechanism involves using thermal energy under different atmospheres to selectively remove surface contaminants, introduce or eliminate oxygen-containing functional groups, and modulate the surface microstructure. For instance, Kim et al. systematically studied the effects of thermal treatment in nitrogen (inert atmosphere) and oxygen (reactive atmosphere) on CF properties. The study demonstrated that moderate thermal treatment (300–500 °C) in an oxygen atmosphere is an effective method for enhancing the interfacial activity of CFs through “surface oxidative functionalization”, particularly suitable for the surface regeneration and performance recovery of recycled CFs. However, temperature and time must be strictly controlled to avoid degradation of the fiber bulk at excessively high temperatures (Fig. 3c) [120].

Beyond conventional methods such as ozone oxidation and thermal treatment, CVD has emerged as a highly effective surface modification technique for inorganic fibers. By introducing gaseous precursors that undergo chemical reactions on the fiber surface, CVD enables precise control over surface composition and microstructure, thereby significantly enhancing interfacial compatibility with metal matrices and improving electrochemical performance. The core mechanism involves the in situ growth of functional coatings with tailored morphology and chemical properties through careful regulation of reaction temperature, pressure, and precursor types. For instance, Liu et al. employed plasma-enhanced chemical vapor deposition (PECVD) to directly grow vertical graphene (VG) on a GF separator surface using CH_4_ as the carbon source at a relatively low synthesis temperature. Subsequent air plasma treatment introduced oxygen and nitrogen heteroatoms, successfully transforming the originally electrochemically inert GF surface into a zinc-affine functional interface. This modification significantly enhanced its affinity toward zinc ions and improved its ability to guide uniform zinc deposition (Fig. 3d) [121].

#### “Wet” Chemical Modification

“Wet” processing involves treatments conducted in liquid media (typically aqueous solutions, sometimes organic solvents), relying on chemical reactions in the liquid environment to alter the physical and chemical properties of the fiber surface, including liquid-phase oxidation [122], electrochemical oxidation [123], sizing, coating [124], and chemical grafting functionalization [125–127].

**Fig. 4:**
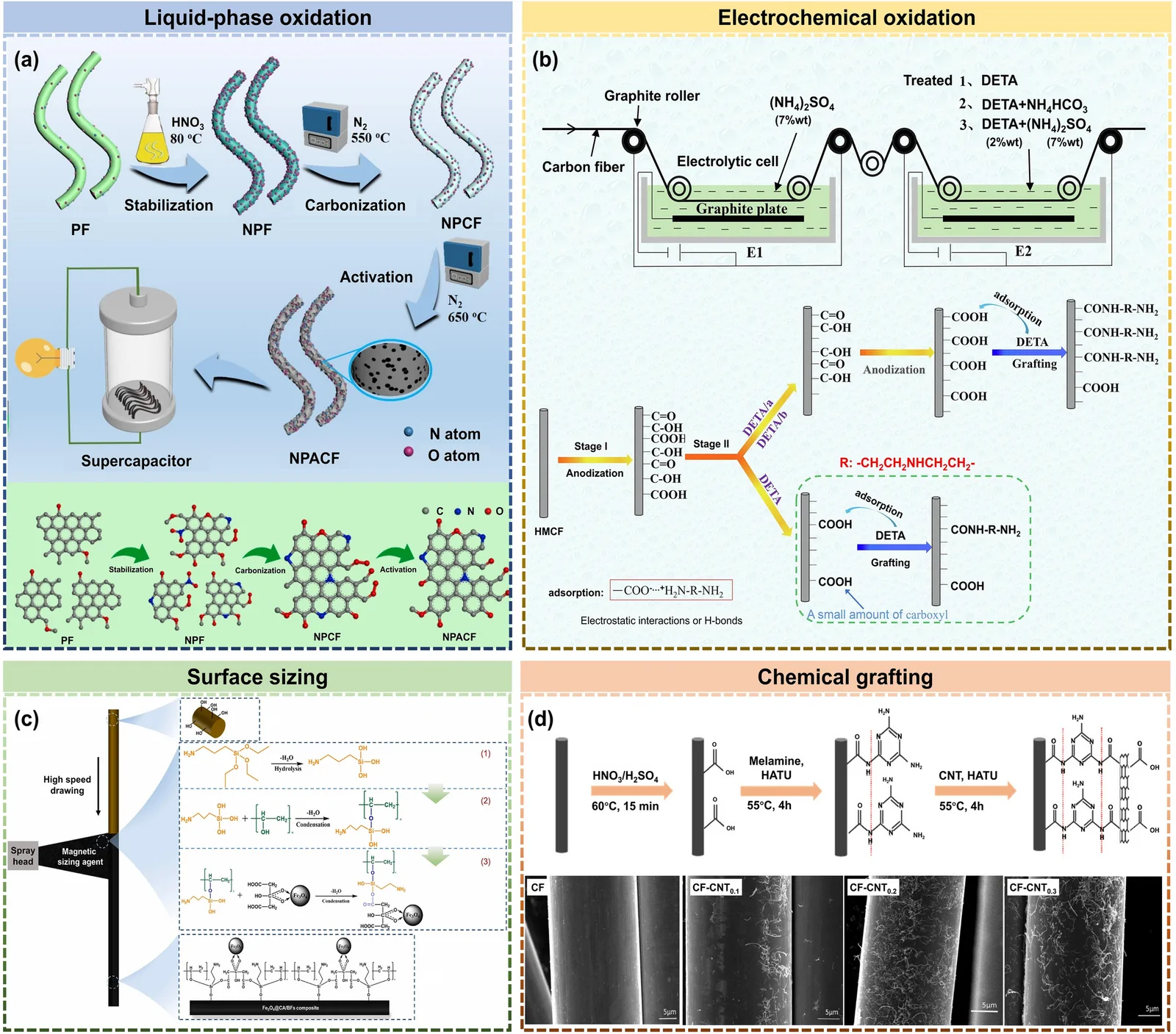
“Wet” chemical modification method for the inert surface of IHPFs: liquid-phase oxidation; electrochemical oxidation; surface sizing; chemical grafting. **a** Preparation of NPACF; Molecular structural evolution. Reproduced with permission from [128]. Copyright 2024, Elsevier B.V.; **b** Process for electrochemical oxidation-grafting treatment; The reaction mechanism on the surface of HMCF with different treatment methods. Reproduced with permission from [129]. Copyright 2020, Elsevier B.V.; **c** Preparation of BFs, Fe_3_O_4_@CA magnetic sizing agent and Fe_3_O_4_@CA/BFs composite. Reproduced with permission from [130]. Copyright 2025, Elsevier B.V.; **d** Chemically grafted modified CFs with CNTs; SEM images of the surface morphologies of CFs. Reproduced with permission from [132]. Copyright 2020, MDPI

Liquid-phase oxidation is a classic “wet” surface modification technique [21]. Its core mechanism leverages the chemical oxidizing power of strong acids (e.g., nitric acid, sulfuric acid) or strong oxidant solutions to efficiently introduce polar functional groups (e.g., oxygen- or nitrogen-containing groups) onto the fiber surface in a liquid environment, while slightly etching the surface to increase specific surface area, thereby significantly improving the fiber’s interfacial compatibility and electrochemical activity. Ni et al. achieved a synergistic effect of “functional group introduction” and “pore structure optimization” through liquid-phase oxidation, ultimately endowing nitrogen-doped pitch-based activated CFs (NPACF) with exceptional electrochemical performance in KOH electrolyte. This study demonstrated that liquid-phase oxidation is an efficient, low-temperature, and rapid strategy for fiber surface activation and functionalization, particularly suitable for low-softening-point precursors that cannot withstand high-temperature treatments, providing critical technical support for preparing inorganic high-performance functional fibers (Fig. 4a) [128].

Electrochemical oxidation is an efficient and controllable wet surface treatment technology. Its core mechanism involves using an applied electric field to drive electrochemical reactions of anions (e.g., OH^−^, NO_3_^−^) or water molecules from the electrolyte on the fiber surface, thereby efficiently and uniformly introducing oxygen-containing functional groups and creating nanoscale rough structures, fundamentally enhancing the interfacial bonding performance with resin matrices. For instance, Fu et al. subjected high-modulus CF (HMCF) surfaces to anodic oxidation via electrochemical oxidation treatment, followed by electrochemical grafting of diethylenetriamine. This simultaneously increased the content of oxygen- and nitrogen-containing functional groups on the HMCF surface, significantly improving the interfacial properties of HMCF composites. The interlaminar shear strength (ILSS) of HMCF/epoxy composites reached 97.5 MPa, a 257.1% increase compared to untreated HMCF (Fig. 4b) [129]. Therefore, electrochemical oxidation is considered a highly promising fiber surface activation technology, especially applicable in high-performance composite fields demanding extreme interfacial properties, effectively addressing the interfacial bonding challenges posed by the surface inertness of high-modulus CFs.

Sizing is a “wet” post-treatment technology widely used in industrial production. Its core mechanism involves passing surface-activated fibers through a sizing agent containing polymer film formers, coupling agents, and functional nanoparticles to form a thin, uniform polymer coating on the surface. This coating not only protects the fibers from abrasion but, more crucially, acts as a bridge, generating strong physical adsorption and chemical bonding with both the fiber surface and the resin matrix through its own functional groups, thereby significantly enhancing the composite's interfacial properties. Li et al. innovatively introduced Fe_3_O_4_ magnetic nanoparticles into a traditional epoxy-based sizing agent. This treatment aimed not only to impart magnetism to BFs but also significantly enhanced their interfacial bonding capacity, fundamentally solving the weak interfacial adhesion caused by the inert surface of BFs. Results showed that both the ILSS and IFSS of the composites were markedly improved after this functional sizing treatment. Additionally, the treatment successfully endowed the fibers and their composites with additional magnetic responsiveness, demonstrating the great potential of sizing technology in achieving integrated “interface strengthening–functionalization” of fibers (Fig. 4c) [130].

Chemical grafting is a “wet” treatment technology that covalently bonds functionally specific molecules or nanomaterials firmly to the fiber surface [131]. It offers strong design flexibility, mild and controllable reaction conditions, and minimal damage to the fiber’s mechanical properties. For instance, Ji et al. vertically and uniformly grafted CNTs onto the CF surface by multistep chemical reactions. This structure increased the fiber’s specific surface area, providing numerous sites for mechanical anchoring. Furthermore, the grafted CNT network can impart additional functionalities like electrical and thermal conductivity to the composites (Fig. 4d) [132]. Compared to methods like physical coating, chemical grafting via covalent bonds offers durable and stable modification effects that resist debonding under high temperatures or shear forces during processing. It represents one of the most powerful technical pathways for achieving a “qualitative leap” in the interface of fiber composites, particularly suited for cutting-edge applications in aerospace and defense where extreme interfacial performance is required.

## Preparation Strategies for EMI Shielding Inorganic High-Performance Fibers/Fabrics

**Fig. 5:**
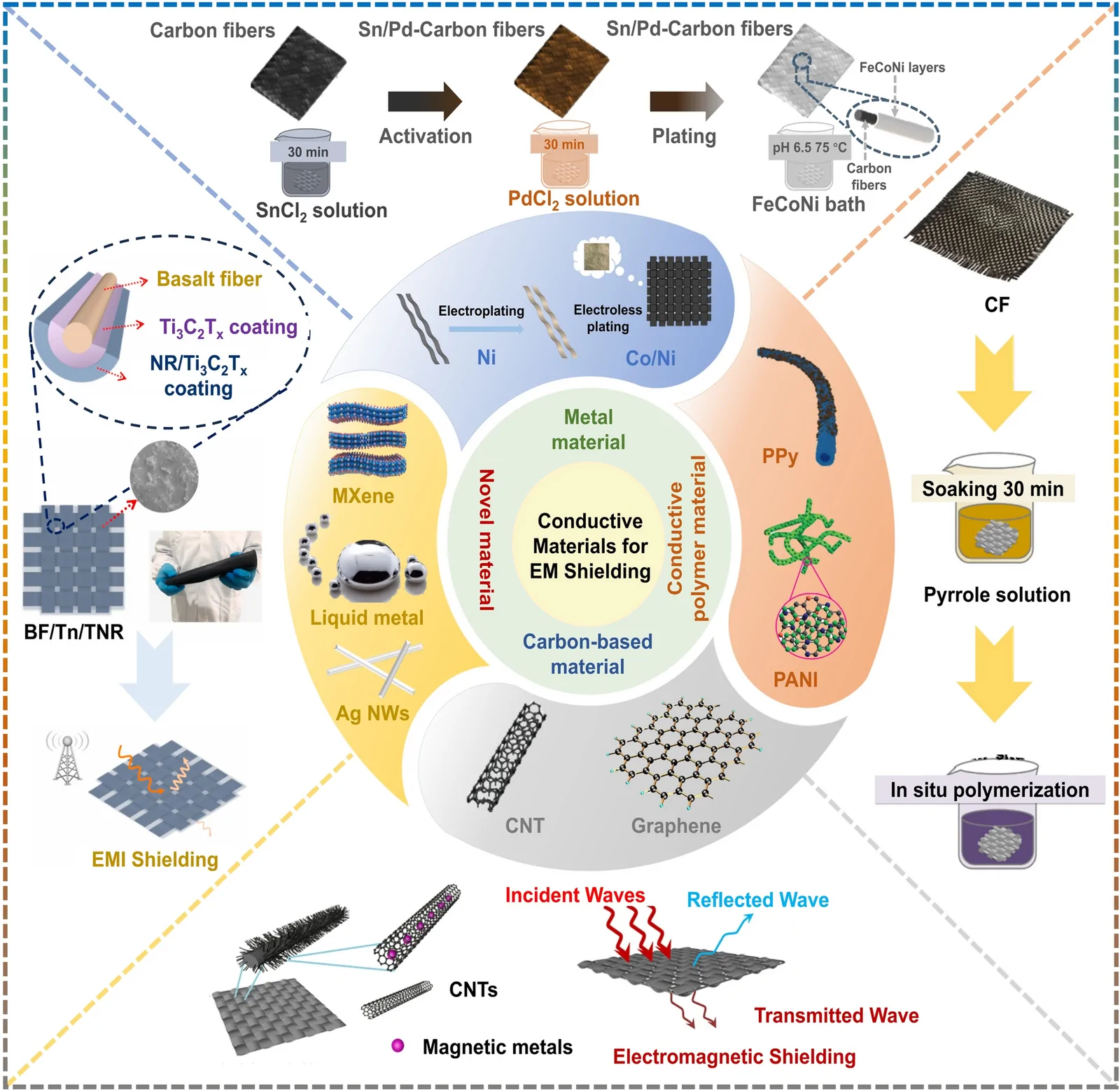
Conductive materials for constructing the EM shielding functional layer: metals (such as Ni, Co/Ni), conductive polymers (such as PPy, PANI), carbon-based materials (such as CNT, Graphene), new materials (such as MXene, liquid metals (LMs), and AgNWs). Reproduced with permission from: [146] Copyright 2021, Elsevier B.V.; [156] Copyright 2021, MDPI; [177] Copyright 2023, Elsevier B.V

IHPFs, owing to their exceptional tensile strength, high modulus, and outstanding environmental resistance, serve as ideal substrates for constructing lightweight and durable EMI shielding materials. However, as previously discussed, their inherent surface chemical inertness and smooth physical morphology severely restrict the robust loading and uniform construction of functional coatings, presenting a primary bottleneck for practical applications. After successfully introducing active sites and improving wettability through surface activation treatments (e.g., plasma, ozone oxidation, and chemical etching), the core research focus shifts to employing precise functionalization strategies to endow these fibers with efficient EMI shielding and absorption capabilities. The key to achieving high-performance EMI shielding lies in constructing a continuous conductive network on the fiber surface and within the bulk phase through precise preparation processes to enhance EM wave reflection loss, while simultaneously introducing abundant magnetic/dielectric loss units, such as metals [133–136], conductive polymers [137–139], carbon-based materials [140–143], and novel materials [144–146] (Fig. 5). This enhances polarization relaxation and energy conversion, thereby promoting the absorption and dissipation of EM waves. To realize this goal, researchers have developed a series of physical and chemical preparation methods (Table 4). Based on their principles and characteristics, these methods can be primarily categorized into physical deposition, chemical treatment, and other emerging technologies. This section will systematically review the principles and processes of these preparation strategies, delve into how different methods regulate the EM parameters and microstructure of the materials, and ultimately achieve customizable EM protection functions, transitioning from “reflection-dominated” to “absorption-dominated” mechanisms.

**Table 4:** Summary of preparation strategies and key parameters (mechanical property, SSE/t, and other indicators) for the EMI shielding functional layer of IHPFs

Preparation method		Substrate	Materials	Frequency (GHz)	EMI SE (dB)	Temperature	Main cost	Key parameters	References
Physical Deposition	Spray-drying	GF	AgNWs; ScPEG	8–12	40–72	Room temp. (spray)	Materials (precursor); energy for drying	Tensile strength (up to 191 MPa)	[157]
		GF	MWCNT; xGnP	0.03–1.5	35.3–56.8	Room temp. (spray)		Thickness: 3.02 ± 0.03 mm	[154]
		BF	Ti_3_C_2_T_x_	8–12	41.53	45 °C (extract); 80 °C (dry)		Electro-thermal property: (4 V, 70 °C)	[144]
		CF	Cu	0.9–1.5	59.8	60 °C (etching)		–	[37]
	Vacuum deposition	CF	CNT	8–12	24.6	50 °C (hot-press)	Equipment; target materials	SSE/t:12,504 dB cm^2^ g^−1^	[164]
Chemical treatment	Electroless plating	GF	Cu	8–12	74.59	20–60 °C (deposition); 60 °C (dry)	Materials; wastewater treatment	Cu layer: 8.02 μm	[169]
		GF	Ni; CNTs	8–12; 1–18	> 50; > 35	45 °C (deposition)		CNTs: 9.2 wt%	[168]
		ACBF	Co–Ni alloy	0.03–6	42.57	80 °C (deposition)		Tensile strength (72.9 MPa)	[172]
		CF	Ni-W-Cu-P	8–12	37	75 ± 2 °C (deposition)		Tensile strength (538.8 MPa)	[40]
		CF; Graphene fiber	Ni; Ag	8–12	33.7	Ag: 40 °C, Ni: 60 °C (deposition)		Thickness: 2 mm; Absorption (82.7%)	[167]
	Electroplating	CF	Ni	8–12	71	25 °C (deposition)	Energy; Environmental cost	Thickness: 1.2 mm; Tensile strength (22.4 MPa)	[173]
		CF	Ni	8–12	23.5–31.6	50 °C (deposition)		Flexural strength (21.1% increase)	[43]
		CF–RGO	Ni	2–18	> 10	–		Thickness:2 mm	[42]
	In situ polymerization	CF	PDA, BN	8–12	50.06	60 °C	Equipment; energy; materials	ILSS and mode II toughness (35.50%, 97.35% increase)	[179]
		CoNi/CF	PPy	2–18	68.78	Room temp		EAB: 5.62 GHz; Thickness:2.43 mm	[180]
	CVD	CF	CNT	18–26.5	40–50	900 °C (120 min)	Equipment; energy; materials (precursor gases)	Overpotential: 344 mV	[183]
		BF	CNT	8–12	37.37	800 °C (60 min)		Thickness: 2 mm	[46]
		AF	Graphene	2–18	85	800–1050 °C (1–300 min)		Growth temperature (~ 200 °C lower); Growth rate (~ 3.4 times faster)	[185]
		AF	Graphene	2–18	> 30	1100 °C (30–300 min)		CVD flow: CH_4_ and H_2_	[186]
		AF	Graphene	2–18	46	800–1100 °C (250 min)		CVD flow: C_3_H_8_ and H_2_	[189]
		AF	Graphene	8–12	25	1000 °C (30 min)		Absorption: > 90%	[117]
	Thermal treatment	Graphene fiber	–	8–12	> 120 (2L)	600–2800 °C (60 min)	Equipment; energy; heating time; gas purity	8.5 × 10^5^ S m^−1^	[191]
		CF	CNT (ZIF–8@ZIF–67)	2–18	> 20	920 °C (pyrolysis)		EAB: 4.4 GHz; Thickness: 1.38 mm	[192]
		CF	CNT(ZIF-L)	8–12	38.4	400 °C (2 h); 900 °C (6 h)		7.50 W m^−1^ K^−1^	[193]
Others	ALD	CF	Al_2_O_3_ + TiO_2_ layers	8–12	45	150 °C	Equipment; materials (precursor)	Structure color	[198]
	Laser etching	BF	Graphene	8–12	~ 20 (1 L); ~ 50 (3 L)	Localized high temp	Materials; safety management	–	[200]
	Dipping	CF	MWCNT	2; 2.7	37; 68	Room temp	Materials	SSE/t: 35,000 dB cm^2^ g^−1^	[203]

### Physical Deposition Method

With the rapid development of electronics in industrial, military, and aerospace sectors, there is a pressing need for adjustable and durable EMI shielding textiles possessing excellent mechanical properties. Physical deposition methods (such as spray-drying, vacuum deposition, and magnetron sputtering) typically involve the physical attachment and film formation of vapor or liquid-phase precursors on the fiber surface. These methods offer advantages including relative process simplicity, minimal damage to the fiber substrate, and ease of scalability [147–149]. Simultaneously, they enable the uniform deposition of conductive or magnetic material coatings on the fiber surface, significantly enhancing its EMI shielding performance. By adjusting deposition parameters (e.g., deposition rate, atmosphere pressure, and energy input), one can not only optimize the coating's density and adhesion but also impart excellent stability and durability to the fiber fabric. Consequently, physical deposition has become an important and widely applied strategy for modifying IHPFs for EMI shielding.

#### Spray-Drying

**Fig. 6:**
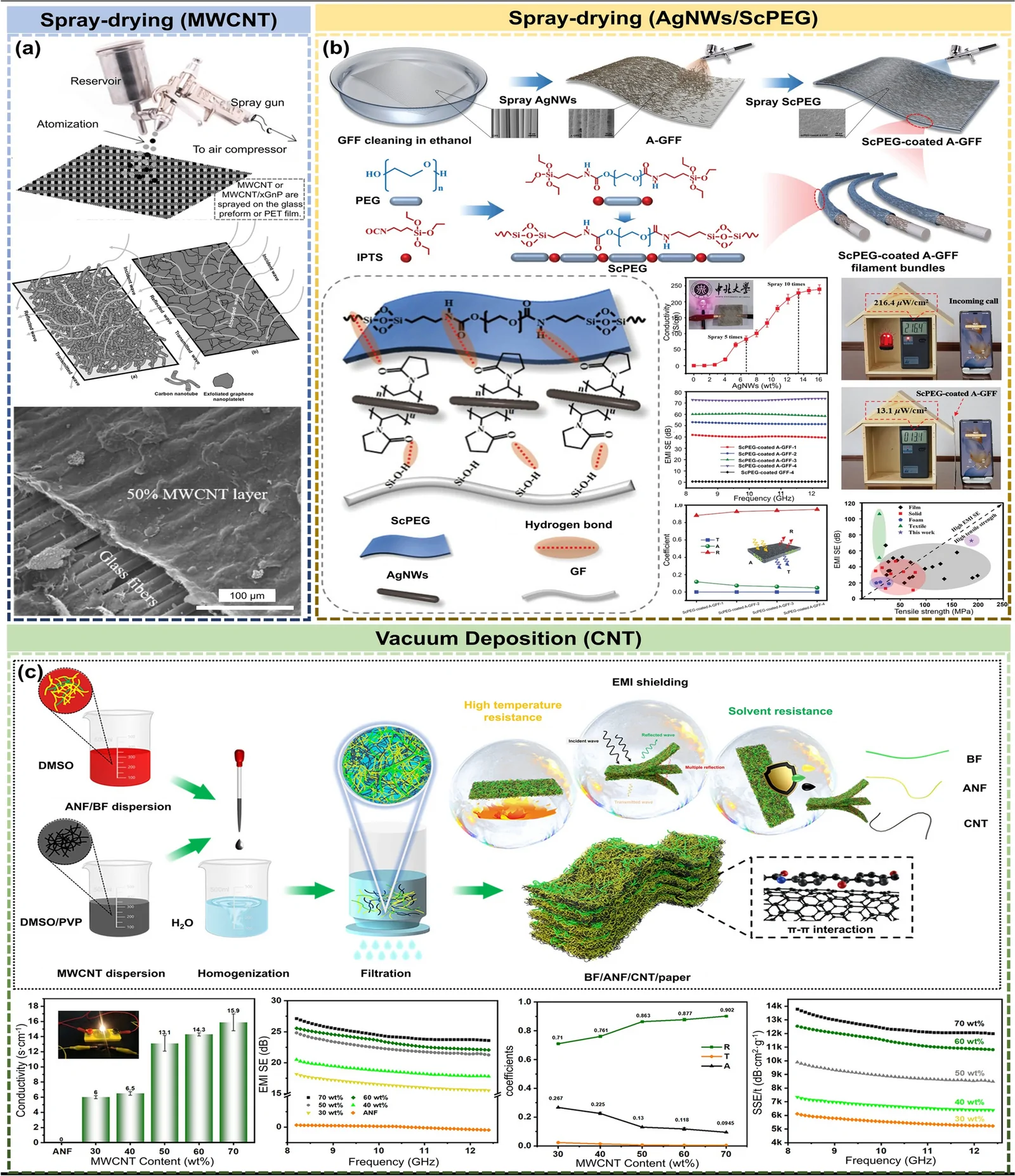
Physical deposition method: spray-drying and vacuum deposition, are used to fabricate EMI shielding functional layers. **a** MWCNT or MWCNT/xGnP are sprayed on the GFs; Illustration of EM waves interactions with CNTs and xGnPs; SEM micrographs of hybrid MWCNT/xGnP coated GFs. Reproduced with permission from [156]. Copyright 2019, Elsevier B.V.; **b** Fabrication of the ScPEG-coated A-GFF; Hydrogen bonds among ScPEG coatings, silver nanowires (AgNWs), and fabric; The conductivity as a function of AgNWs content with different spraying times; EMI SE and power coefficient of ScPEG-coated A-GFF; EMI shielding mechanism. Reproduced with permission from [159]. Copyright 2024, Elsevier B.V.; **c** Fabrication process of BF/ANF/CNT composite paper; Electrical conductivity, EMI SE, power coefficient, and SSE/t of BF/ANF/CNT composite paper. Reproduced with permission from [166]. Copyright 2023, John Wiley & Sons

Spray-drying is a relatively mature technology characterized by low cost, simple operation, and high productivity compared to other techniques. These advantages make it particularly suitable for fabricating conductive metal structures on flexible substrates and for large-scale manufacturing [150, 151]. In EMI shielding composite research, multiwalled carbon nanotubes (MWCNTs) [152, 153] and exfoliated graphite nanoplatelets (xGnPs) [154, 155] are widely used to construct efficient conductive networks due to their excellent electrical conductivity and unique micro-morphology, significantly enhancing the material’s EMI SE. As shown in Fig. 6a, Park et al. utilized a spray gun to coat a mixed suspension of MWCNTs and xGnPs onto a GF surface, followed by resin infiltration and molding to prepare a multilayer composite shielding material. By optimizing parameters such as MWCNT length, xGnP size, mixing ratio, and number of coating layers, the system enhanced conductive pathways and interfacial structure, ultimately achieving a shielding performance of 35.3–56.8 dB in the frequency range of 30 MHz to 1.5 GHz [156]. The spray-molding process demonstrates good scalability and applicability, laying the foundation for developing multifunctional composites that integrate EMI shielding, self-sensing, and structural intelligence. In the field of EMI shielding composites, AgNWs [157, 158] have become an important material for constructing highly efficient conductive networks due to their high conductivity, large aspect ratio, and excellent mechanical flexibility, making them especially suitable for flexible fabric-based functional composites. As shown in Fig. 6b, Liang et al. modified a GF fabric (GFF) surface with AgNWs via spray-drying and further coated it with a self-cross-linking solid–solid phase change polyethylene glycol (ScPEG) layer based on multiple hydrogen bond cross-linking. The single-layer ScPEG coating A-GFF revealed the excellent and practical function of blocking radio wave transmission of the sample by the change of the indicator light in the safe house. This successfully produced an integrated fabric composite with excellent EMI shielding, thermal management, and information encryption functionalities, offering new ideas for the multiscenario application of high-end EM protection structures in aerospace, military equipment, and smart buildings [159]. Compared to precious metals like silver and gold, copper offers advantages such as lower processing cost, high conductivity, and good ductility, making it a common metallic coating material for enhancing the EMI shielding performance of fiber-based composites [160–162]. Chen et al. used a spray-assisted deposition method to construct a nanostructured copper coating on a CF fabric (CFF) surface. By controlling the number of spraying cycles (10 to 100 cycles), they achieved uniform coverage of a flocculent hierarchical rough structure composed of Cu nanoparticles, which exhibited excellent EMI shielding performance. The SE_T_ reached approximately 61.3 dB, nearly 20 dB higher than that of untreated CFF. Although the stability of this material under high-temperature and salt spray environments requires further improvement, this study demonstrates the good application potential of spray-assisted deposition technology for preparing lightweight, high-performance flexible EMI shielding textiles [37].

#### Vacuum Deposition

Vacuum deposition is a physical vapor deposition technique conducted in a vacuum or high vacuum environment, where evaporated or sputtered material is deposited onto the fiber surface. This method effectively reduces interference from gas molecules during the deposition process, resulting in coatings with higher purity and better density [163–165]. By adjusting parameters such as deposition rate, vacuum level, and substrate temperature, the thickness, crystal structure, and adhesion of the coating can be controlled, enabling precise tuning of SE and durability. Furthermore, as this method does not significantly affect the mechanical properties of the fiber, it is considered an effective approach for the surface functionalization of high-performance fibers. As shown in Fig. 6c, Song et al. used BF as the skeleton, aramid nanofiber (ANF) as the reinforcement, and CNTs as the conductive network. They successfully constructed a BF/ANF/CNT composite paper with a layered structure via vacuum-assisted filtration technology. In the X-band (8.2–12.4 GHz), this material, with a thickness of 48 μm, achieved a SE_T_ of 24.6 dB. Its SSE per unit thickness (SSE/t) was as high as 12,504 dB cm^2^ g^−1^, demonstrating exceptional EMI shielding performance, environmental stability, and Joule heating performance [166]. The material not only performs excellently under conventional conditions but also maintains structural and functional integrity under extreme conditions, showing broad application prospects. This work provides new ideas for addressing the reliability issues of EMI shielding materials in extreme environments and offers important references for the design and preparation of multifunctional integrated flexible electronic materials.

### Chemical Treatment Method

Compared to physical deposition methods, chemical treatment techniques construct functional coatings on fiber surfaces through chemical reactions or modulation of the chemical environment, offering advantages such as strong adhesion, continuous film formation, and multiscale interfacial control. Typical methods include electroless plating, electroplating, in situ polymerization, chemical vapor deposition/infiltration, and annealing treatments. Chemical treatments can introduce highly conductive or magnetic functional layers onto the fiber surface, while effectively regulating coating thickness, microstructure, and interfacial adhesion by adjusting reaction conditions (e.g., solution concentration, temperature, reaction time, and catalyst type) [167]. These methods not only endow IHPFs with excellent EMI SE but also balance flexibility, durability, and stability in complex environments, thereby holding broad application potential in the preparation of EMI shielding fiber composites.

#### Electroless Plating

**Fig. 7:**
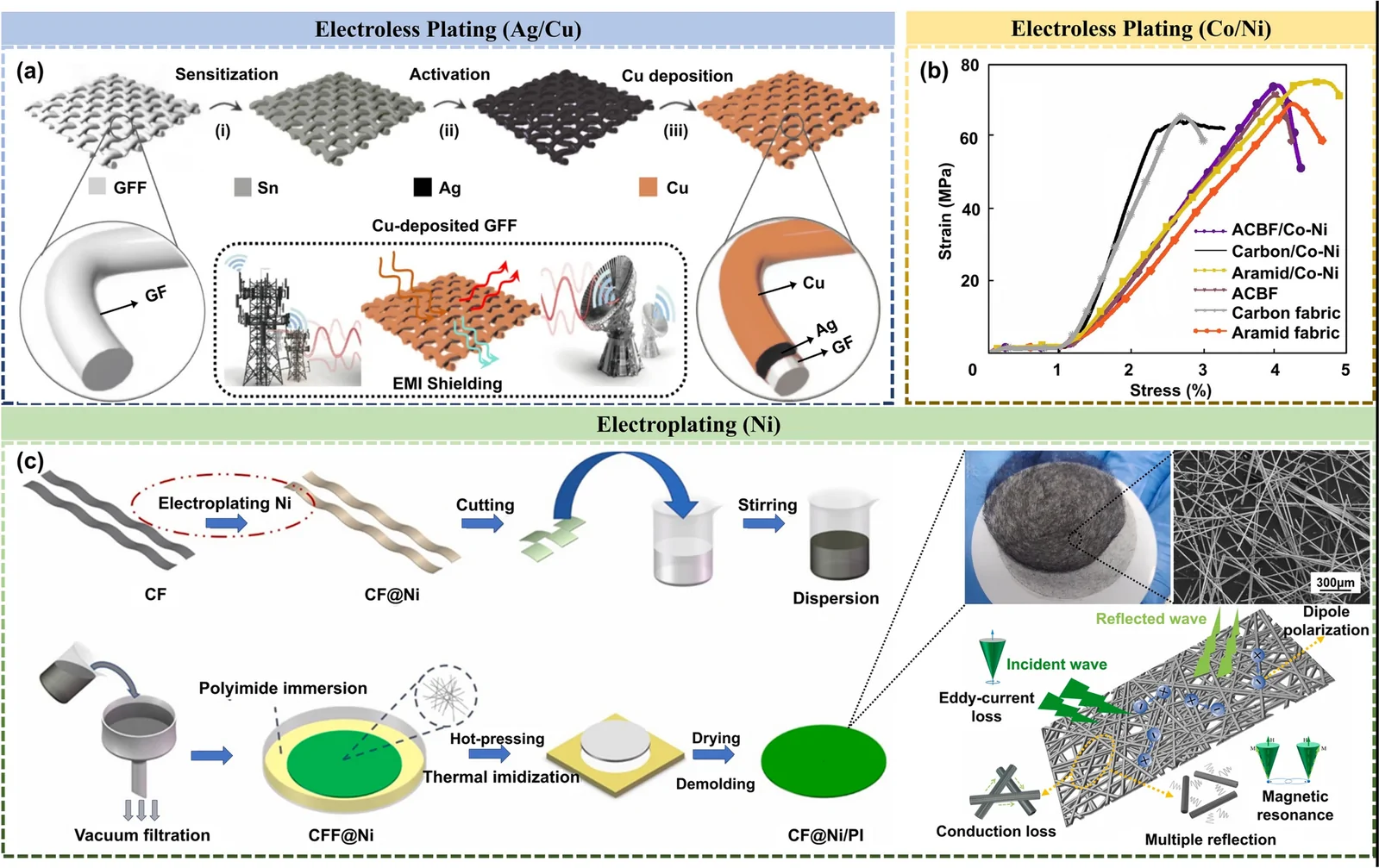
Fabrication for EMI shielding functional layers by electroless plating and electroplating method. **a** Fabrication process for electroless Cu deposition on GFF. Reproduced with permission from [173]. Copyright 2025, Elsevier B.V.; **b** Stress–strain curves of ACBF/Co–Ni and other samples (The intrinsic toughness of Co–Ni alloy coating enhances the tensile strength of ACBF/Co–Ni.). Reproduced with permission from [176]. Copyright 2021, Elsevier B.V.; **c** Fabrication process of the proposed composite film; Optical and SEM images of CFF@Ni; EM shielding behavior of CF@NN/PI. Reproduced with permission from [177]. Copyright 2023, Elsevier B.V

Electroless plating is an autocatalytic reduction process that does not require an external power source, enabling the uniform deposition of conductive metal layers on fiber surfaces [168, 169]. Its advantage lies in achieving continuous coverage on fibers or fabrics with complex morphologies, with coating thickness controllable via reaction time and solution composition. Commonly used metals include Ni, Cu, and Ag, which significantly enhance the electrical conductivity and EMI shielding performance of fibers [170–172]. Simultaneously, the coatings formed by electroless plating exhibit strong adhesion to the substrate, imparting excellent durability and stability to the fibers, making this method widely applicable in the construction of EMI shielding textiles. Copper electroless plating, due to its mature technology, low cost, and ease of scalability, is extensively used to build continuous metallic conductive layers on fiber surfaces, particularly for the EMI shielding functionalization of IHPFs like GFs. As shown in Fig. 7a, Parkash et al. achieved continuous and dense copper layer coverage on the inert surface of GFs via electroless copper plating, with a SE_T_ of 74.59 dB in the X-band, significantly superior to that of untreated GFF (SE_T_ = 1.10 dB). They systematically investigated the effect of deposition temperature (20–60 °C) on the microstructure, electrical conductivity, and EMI shielding performance of the electroless copper layer on GFF [173]. By precisely controlling the deposition temperature, the crystallinity, thickness, and density of the copper layer could be regulated, enabling the production of high-performance, lightweight, and flexible EMI shielding textiles without complex pretreatment.

Beyond single-metal coatings, alloy electroless plating also demonstrates significant advantages in enhancing the EMI shielding performance of fiber-based composites due to its tunable multifunctional properties [174, 175]. Wang et al. successfully constructed a cobalt–nickel (Co–Ni) alloy coating on the surface of an aramid-carbon blended fabric (ACBF) using electroless plating, markedly improving the material’s EMI SE and mechanical strength. The average SE_T_ reached 42.57 dB in the 30–6000 MHz frequency range, primarily attributed to the synergistic effect between the dielectric loss of CFs and the magnetic loss of the Co–Ni alloy, achieving dual attenuation of EM wave absorption and reflection. Furthermore, the material exhibited excellent corrosion resistance and high mechanical strength, showing broad application prospects in fields such as aerospace flexible electronic equipment and military protective textiles (Fig. 7b) [176]. This method features a mild process that avoids strong acids or alkalis and can be completed within 2–3 h, demonstrating good potential for industrialization. Electroless coatings endow IHPFs with high conductivity, forming continuous conductive networks responsible for reflection-dominated EMI shielding. In addition, the in situ redox deposition process facilitates strong chemical bonding between the coating and the fiber surface, significantly enhancing interfacial adhesion and mechanical integrity.

#### Electroplating

Electroplating is a method that uses an external electric field to drive the reduction and deposition of metal ions onto fiber surfaces, enabling the acquisition of highly dense and pure metal coatings in a relatively short time. Compared to electroless plating, electroplating offers higher controllability over deposition rate and thickness and is suitable for large-area continuous processing. By adjusting process parameters such as current density, electrolyte concentration, and temperature, the adhesion and microstructure of the coating can be effectively improved, thereby enhancing EMI shielding efficiency. However, electroplating typically requires the fiber substrate to possess some conductivity, so it is often combined with other surface activation or pretreatment methods. As shown in Fig. 7c, Wang et al. used electroplating to construct two types of nickel coatings on CF surfaces: conventional flat and nanocone-shaped (denoted as CF@CN and CF@NN, respectively). They found that differential adsorption of NH_4_^+^ ions in the plating solution induced the growth of nickel grains along specific orientations, forming nanocone array structures approximately several hundred nanometers in height (CF@NN). This structure not only enhanced the contact probability and mechanical interlocking effect between fibers but also significantly improved the electrical conductivity (41.2 S m^−1^) and EMI SE_T_ (71 dB in the X-band at a thickness of only 1.2 mm) of the composite film. Additionally, the CF@NN/PI composite film exhibited excellent mechanical properties (tensile strength of 22.4 MPa, elastic modulus of 90 MPa) and flexibility, withstanding repeated bending without fracture, indicating broad application prospects in microelectronic devices and flexible wearable systems [177].

#### In Situ Polymerization

**Fig. 8:**
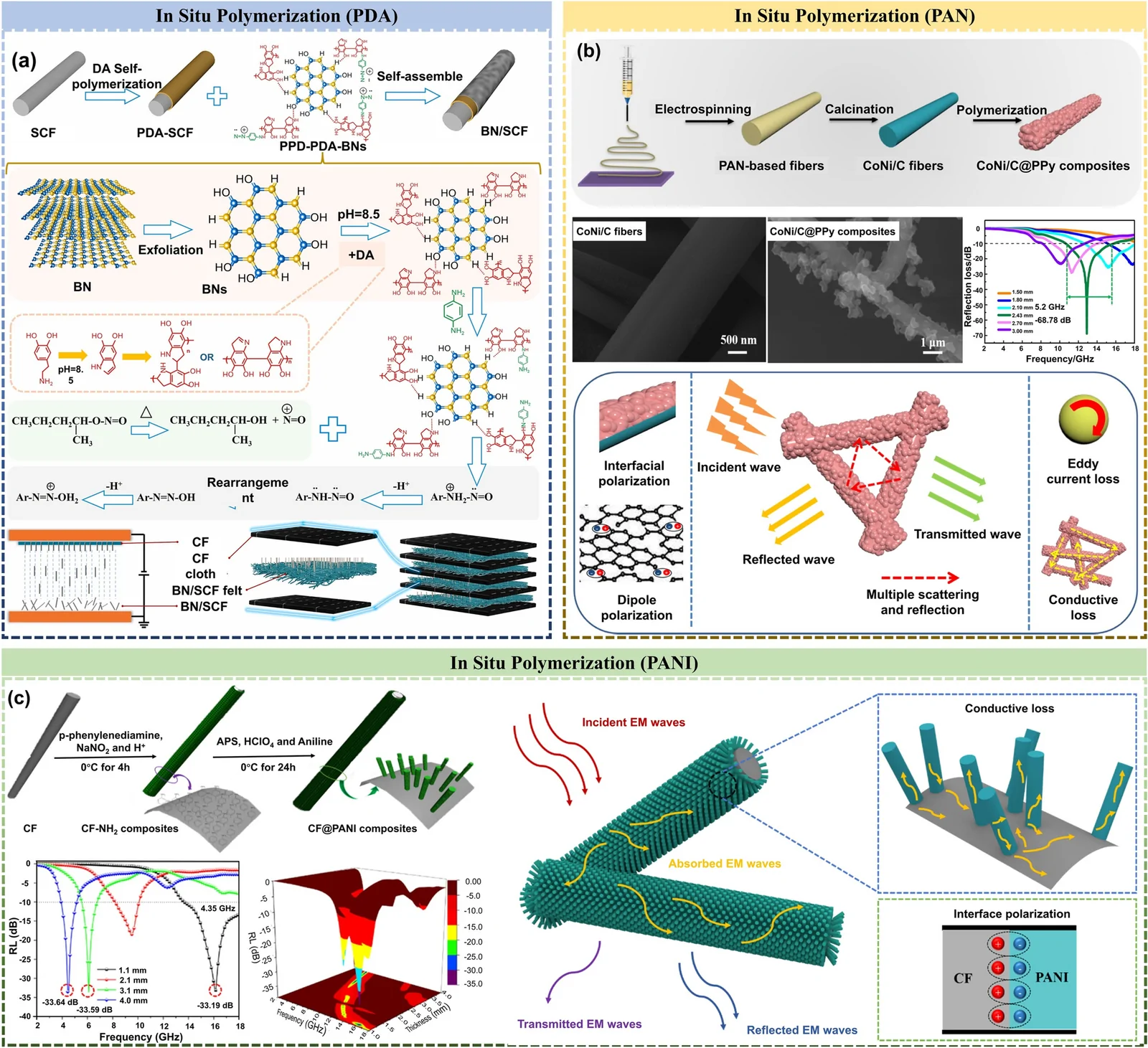
Fabrication for EMI shielding functional layers by in situ polymerization method. **a** Preparation of BN/SCF; Preparation of PPD-PDA-BNs; Preparation of BN/SCF felt-x/CF/EP composites. Reproduced with permission from [183]. Copyright 2025, Elsevier B.V.; **b** Synthesis processes of CoNi/C@PPy composites; SEM images of CoNi/C fibers and CoNi/C@PPy composites; RL values of CoNi/C; The possible EM wave absorption mechanisms of CoNi/C@PPy composites. Reproduced with permission from [184]. Copyright 2022, Elsevier B.V.;** c** Synthesis procedure of CF@PANI; RL and 3D plots of CF@PANI composites; EM wave-absorbing mechanisms. Reproduced with permission from [185]. Copyright 2025, Elsevier B.V

In situ polymerization refers to the method of directly initiating monomer polymerization reactions on the surface of fibers or fabrics to form conductive polymer coatings. Common polymers include polypyrrole (PPy) [178], polyaniline (PANI) [179], and polythiophene (PTh) [180]. These conductive polymers not only impart excellent electrical conductivity to fibers but also combine lightweight, flexibility, and good processability. By controlling polymerization conditions (e.g., type of oxidant, reaction temperature, and monomer concentration), the coating thickness, conductivity, and interfacial adhesion can be regulated controllably, making this a strategy that balances performance and process applicability in EMI shielding fibers. It is particularly suitable for constructing polymer coatings that integrate conductivity, dielectric properties, and good interfacial bonding [181, 182]. As shown in Fig. 8a, Luo et al. successfully constructed a core–shell structured boron nitride/short CF (BN/SCF) on the surface of SCF via polydopamine (PDA)-assisted in situ polymerization and electrostatic assembly technology. Using high-voltage electrostatic flocking to achieve oriented alignment, they ultimately prepared a multifunctional CF/epoxy composite via vacuum-assisted resin infusion (VARI). The composite achieved an EMI SE of 50.06 dB, while the ILSS and fracture toughness increased by 35.50% and 97.35%, respectively, providing a new approach for realizing lightweight, high-shielding, and tough integrated composites through in situ polymerization interface engineering [183].

Besides PDA, PPy is another commonly used conductive polymer widely applied in the functional modification of fiber surfaces to enhance their EMI performance. As shown in Fig. 8b, Ma et al. prepared CoNi alloy-embedded CFs (CoNi/C) via electrospinning and carbonization technology, and further coated them with PPy using in situ polymerization to successfully construct CoNi/C@PPy composites. The material exhibited excellent EM wave absorption performance from low frequency to the Ku band, with a minimum reflection loss (RL_min_) of –68.78 dB and an effective absorption bandwidth (EAB) of 5.2 GHz, achieved at a low filler loading of only 15 wt% [184]. Research indicated that the introduction of PPy not only enhanced the conductive loss and interfacial polarization of the material but also significantly optimized impedance matching and attenuation capability through its synergistic effect with CFs and magnetic particles. Furthermore, Gou et al. constructed a core–shell structured CF@PANI composite functional layer by covalently grafting polyaniline nanorods (PANI) onto amino-functionalized CFs (CF-NH_2_) via diazonium reaction and in situ polymerization (Fig. 8c) [185]. The CF@PANI–5 sample exhibited optimal EM wave attenuation capability. Compared with CVD and other vapor-phase deposition techniques, in situ polymerization does not require high-temperature treatment. The resulting polymer layer has moderate electrical conductivity and inherent flexibility. It can not only prevent micro-cracks in the bottom coating during material bending or thermal cycling, but also is suitable for combining with other technologies to construct EMI shielding functional layers that combine reflection and absorption. Looking to the future, optimizing the interfacial chemistry between the polymer and the fiber, the developing green, scalable polymerization methods will be the key to advancing flexible and sustainable EMI shielding materials.

#### Chemical Vapor Deposition

**Fig. 9:**
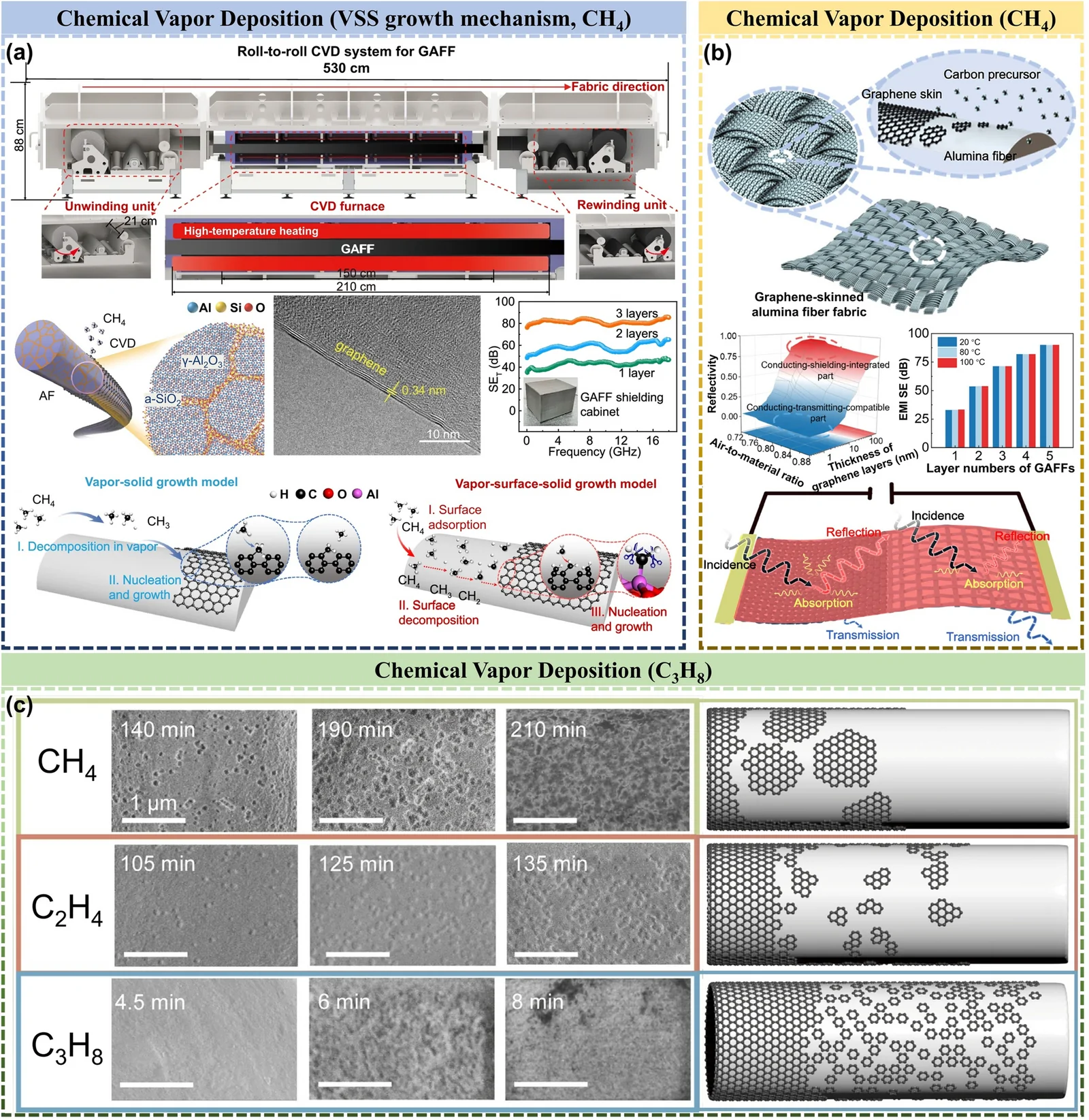
Fabrication for EMI shielding functional layers by CVD method. **a** Mass production of GAFF with the home-made roll-to-roll CVD growth; High resolution-transmission electron microscope (HRTEM) image of three-layer graphene on AF; EMI SE of GAFF; Graphene vapor–solid growth model and graphene vapor surface–solid growth model. Reproduced with permission from [189]. Copyright 2025, John Wiley & Sons.; **b** Preparation of GAFFs via CVD process; EM reflectivity of GAFFs with different air-to-material ratios and graphene coating thicknesses; Adjustable electrothermal-electromagnetic-compatible performances. Reproduced with permission from [190]. Copyright 2025, John Wiley & Sons. **c** SEM images of the graphene domain regions on AF with three carbon sources of CH_4_, C_2_H_4_, and C_3_H_8_; Comparison of the graphene growth behavior of C_3_H_8_, CH_4_, and C_2_H_4_ carbon sources. Reproduced with permission from [193]. Copyright 2025, Tsinghua University Press Ltd

CVD is a method that utilizes gaseous precursors which decompose at high temperatures and deposit onto fiber surfaces. This technique can form inorganic coatings—such as carbon, carbides, or metal oxides—that are structurally dense, uniformly thick, and tightly bonded to the substrate [186–188]. CVD demonstrates outstanding performance in enhancing the thermal stability, corrosion resistance, and EMI shielding properties of fibers. As shown in Fig. 9a, Liu et al. directly grew graphene on the surface of alumina fiber fabric (AFF) using a self-designed roll-to-roll CVD system, successfully preparing graphene-coated alumina fiber fabric (GAFF). The study, for the first time on a non-metallic substrate, revealed a metal-catalysis-like “vapor-surface–solid” (VSS) growth mechanism. Compared to the traditional “vapor–solid” (VS) model followed on conventional non-catalytic non-metallic substrates; this mechanism significantly reduced the graphene growth temperature (by approximately 200 °C) and increased the growth rate (by about 3.4 times). The obtained GAFF not only inherited the high strength, lightweight, and flexibility of the alumina fibers but also exhibited broadly tunable electrical conductivity and excellent EMI shielding performance (SE_T_ up to 85 dB). Furthermore, the self-designed roll-to-roll CVD system enabled the stable large-scale production of GAFF, laying a solid foundation for its practical application in efficient, lightweight EMI shielding composites [189]. Building on this research, Liu et al. further explored the potential of GAFF for multifunctional integration and broad-spectrum performance regulation. By precisely controlling the thickness of the graphene during CVD and the pore structure of the fabric itself, they successfully achieved wide-range adjustment of multiple performance parameters of GAFF, including electrical conductivity, electrothermal temperature, and EM wave reflectivity and transmissivity. This not only enabled the roll-to-roll mass production of GAFF in multiple specifications but also promoted the leapfrog development of graphite-based composites from single function to multidimensional compatibility of “structure–function–environment” (Fig. 9b) [190].

Methane (CH_4_) is commonly used as a carbon source for graphene growth on insulating substrates [191, 192]. Based on their previous work, Liu et al. further explored the feasibility of using propane (C_3_H_8_) for the rapid preparation of graphene on AFF and systematically compared its growth behavior and mechanism with traditional carbon sources (CH_4_, C_2_H_4_). The study found that C_3_H_8_ pyrolysis at high temperatures generates unique active carbon species, C_3_H, which exhibit lower migration energy barriers, higher nucleation density, and faster growth rates on the AFF surface, thereby significantly enhancing the coverage speed and quality of graphene. Compared to CH_4_ and C_2_H_4_, C_3_H_8_ reduced the nucleation time from 110 and 48 min to just 2 min, increased the nucleation density by 160 and 50 times, respectively, and increased the growth rate by more than 10 times, achieving near-complete coverage efficiently within 8 min. The resulting GAFF not only possessed excellent tunable electrical conductivity, high mechanical strength, and flexibility but also showed broad application prospects in EMI shielding (Fig. 9c) [193]. This research provides a new strategy for the efficient, large-area, transfer-free preparation of graphene on non-catalytic insulating substrates, advancing the practical application of graphene in functional composites. Furthermore, for CVD technology, the composition and microstructure of the coating can be precisely regulated by optimizing the reaction atmosphere, temperature, and precursor types, forming carbon nanostructures with hierarchical conductive networks, thereby triggering multiple scattering, dielectric polarization and conductive loss, achieving EMI shielding through both reflection and absorption. The in situ growth process ensures excellent interface contact between the carbon coating and the fiber substrate, forming conductive pathways with excellent interface adhesion and thermal stability. However, the high processing temperature and equipment cost of CVD are still obstacles for large-scale implementation. Future work can focus on improving methods such as low-temperature catalytic growth to achieve a broadband, efficient and scalable EMI shielding system.

#### Thermal Treatment

Thermal treatment is typically employed as a post-processing step following chemical deposition to improve the structure and properties of metal or inorganic coatings. At high temperatures, grains within the deposited layer grow, while defects and interfaces are reduced, thereby enhancing the density and electrical conductivity of the coating. For metal-plated fibers, annealing not only strengthens the adhesion between the coating and the substrate but also reduces interfacial stress, improving its stability and durability [194]. The choice of annealing temperature and time significantly influences the coating’s microstructure and EMI SE; therefore, an appropriate heat treatment process is crucial for achieving high-performance EMI shielding fibers.

**Fig. 10:**
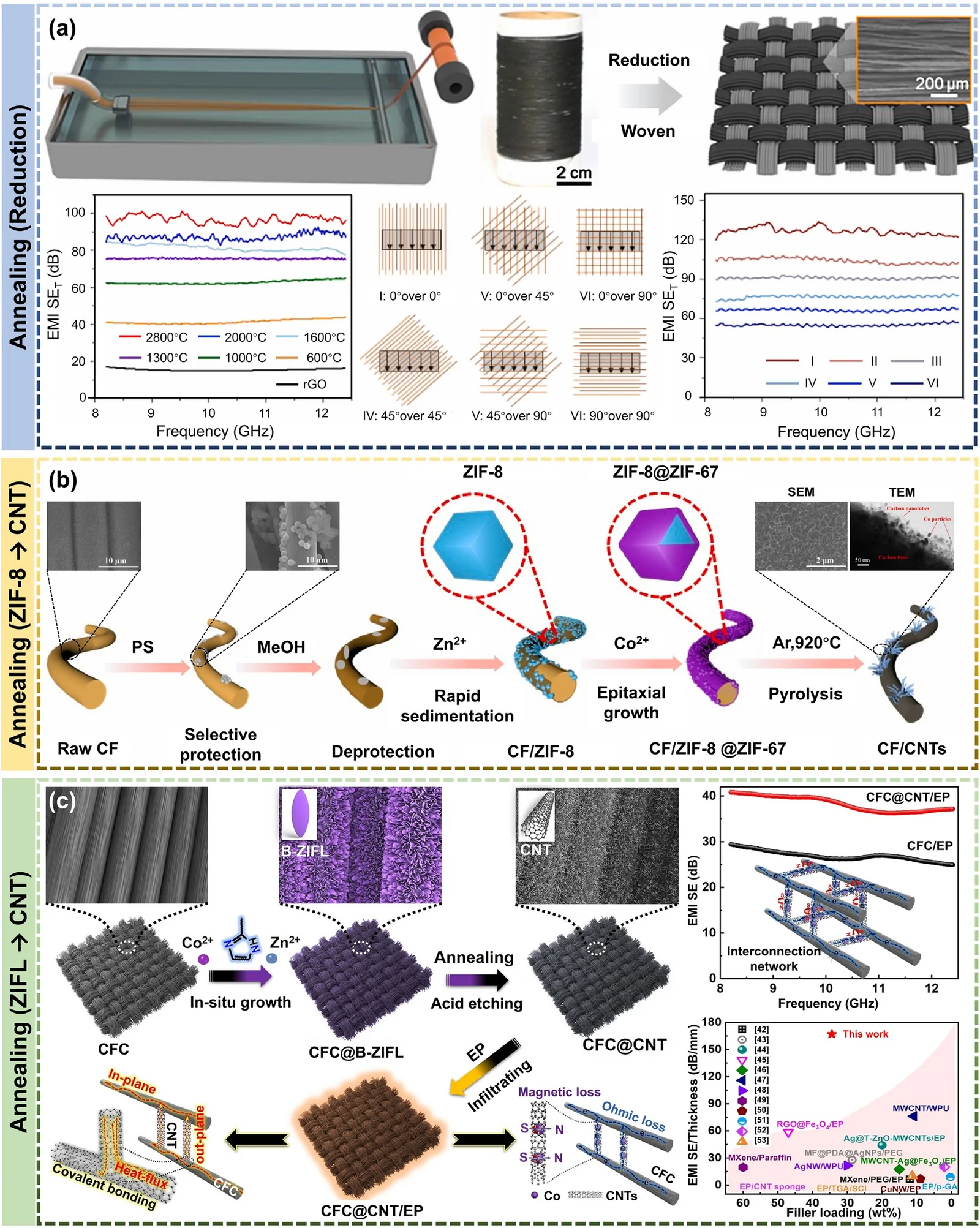
Fabrication for EMI shielding functional layers by thermal treatment method. **a** Preparation and EMI SE of graphene fiber fabrics, and combinations of overlapping two layers of graphene fiber fabrics. Reproduced with permission from [195]. Copyright 2024, Elsevier B.V.; **b** Growth process of CNT cluster arrays on CF (ZIF-8@ZIF-67 transforms into CNTs by pyrolysis). Reproduced with permission from [196]. Copyright 2023, Elsevier B.V.; **c** Synthesis process, isotropic heat transfer, and EMI shielding behavior of CFC@CNT/EP (EMI shielding performance of CFC@CNT/EP is significantly superior to that of existing studies.). Reproduced with permission from [197]. Copyright 2022, American Chemical Society

Furthermore, beyond the strategy of directly growing graphene on substrate surfaces, high-temperature annealing of pre-formed carbon-based fibers has also emerged as a key method for optimizing their intrinsic electrical and EM properties. As shown in Fig. 10a, Gao et al. prepared graphene oxide (GO) fibers via wet spinning and obtained a series of graphene fibers through chemical reduction and high-temperature annealing (600–2800 °C). The heat treatment optimized the structure and significantly enhanced the electrical conductivity of the fibers, increasing from 1.2 × 10^4^ S m^−1^ after chemical reduction to 8.5 × 10^5^ S m^−1^ after annealing at 2800 °C. When woven into fabric, it exhibited exceptional EMI shielding performance in the X-band: the SE_T_ of a single-layer fabric reached up to 96 dB, and by adjusting the texture direction and layering, the SE_T_ of up to 126 dB could be achieved, along with excellent flexibility and durability [195]. This work demonstrates that high-temperature heat treatment is an efficient and scalable strategy for repairing the structure of carbon materials and enhancing their intrinsic shielding performance, providing an important pathway for developing next-generation high-performance flexible EMI shielding textiles.

While directly optimizing the intrinsic conductive and shielding properties of carbon-based fibers through high-temperature heat treatment, another strategy based on the thermal conversion of metal–organic frameworks (MOFs) to derive CNTs offers a new approach for constructing hierarchical structures and achieving efficient, green EMI shielding. Hou et al. developed a novel method for the selective growth of CNT cluster arrays on the CF surface. This method used PS microspheres as a mask to regulate the distribution of surface functional groups on CF. Through bimetallic ZIF-8@ZIF-67 MOF coating and subsequent high-temperature pyrolysis, Co-doped CNT cluster arrays were catalytically grown in specific regions (Fig. 10b) [196]. Research indicated that the density and distribution of the CNT clusters could be precisely controlled by the concentration of PS microspheres and the degree of H_2_O_2_ treatment. In terms of EMI shielding performance, the material’s SE_T_ exceeded 20 dB in the 2–18 GHz range, with a maximum green shielding index (g_s_) of 351 and g_s_ > 12 across the entire frequency band, far superior to the commercial green shielding standard (g_s_ > 1), demonstrating excellent absorption-dominated green shielding characteristics. Moreover, extending this method to integrated thermal management-EMI shielding composites enables multifunctional synergy and performance enhancement. Wu et al. achieved in situ growth of leaf-like bimetallic ZIF-L (Co/Zn) on the surface of CF cloth (CFC) followed by high-temperature conversion, successfully constructing a three-dimensional interconnected network of aligned CFs bridged by carbon nanotubes (CFC@CNT). This was further compounded with epoxy resin (EP) to create a multifunctional composite. In this structure, the ZIF-L-derived CNTs not only effectively connected adjacent CFs, significantly reducing interfacial thermal resistance, but the encapsulated Co nanoparticles also provided stable magnetic loss capability. The resulting CFC@CNT/EP composite exhibits exceptional anisotropic thermal conductivity and high-efficiency EMI SE (38.4 dB). Furthermore, the composite maintains stable mechanical properties, thermal conductivity, and shielding performance even after hundreds of bending cycles, demonstrating outstanding comprehensive application potential (Fig. 10c) [197]. Thermal treatment plays a crucial role in the crystallinity, phase composition, and interface microstructure of each functional layer on IHPFs. Appropriate annealing not only enhances the electrical and magnetic properties of the coating (NiO → Ni) by promoting grain growth and defect repair, thereby regulating the two dominant EMI shielding mechanisms of reflection and absorption, but also improves the interface diffusion and adhesion between the coating and the fiber substrate. However, excessively high temperatures may damage the integrity of the fibers or cause the coating to crack. Therefore, optimizing the thermal treatment scheme and environmental control is crucial for achieving a balance between the material’s electrical conductivity and mechanical stability.

### Other and Emerging Technologies

**Fig. 11:**
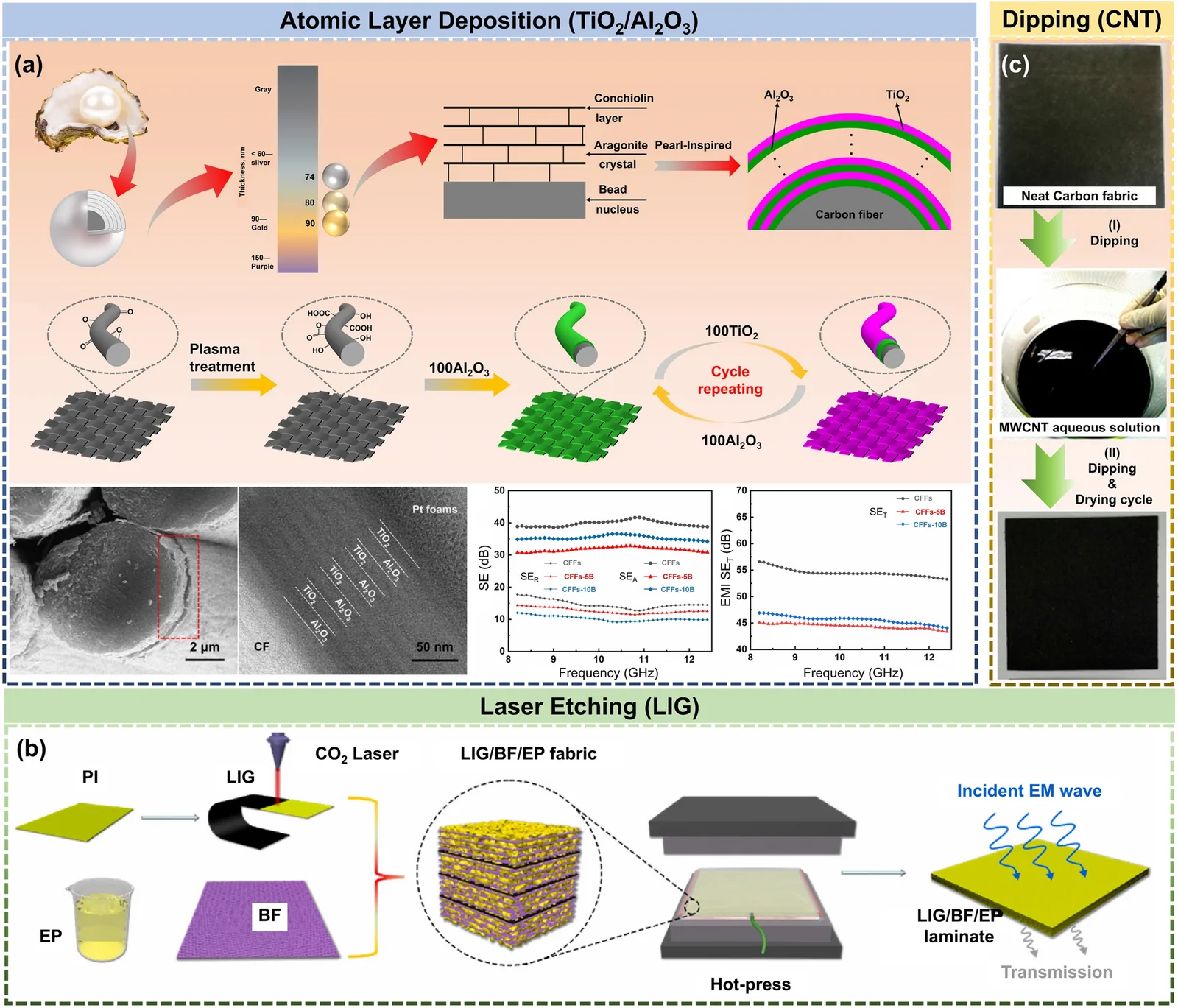
Other and emerging technologies: ALD, laser etching, and dipping methods are used to fabricate EMI shielding functional layers. **a** Schematic illustration, cross section SEM images, and HRTEM image for colored CF with good EMI shielding property through bio-inspired pearls. Reproduced with permission from [202]. Copyright 2025, John Wiley & Sons; **b** Schematic illustration of the fabrication process of laser-induced graphene inserted BF-reinforced epoxy laminates. Reproduced with permission from [204]. Copyright 2023, Elsevier B.V.; **c** C/C composites were manufactured by dip-coating technique, which is suitable for large-scale preparation. Reproduced with permission from [205]. Copyright 2016, Springer Nature

Beyond traditional physical deposition and chemical treatment methods, several emerging technologies have gradually been applied to the surface functionalization of IHPFs in recent years. Novel techniques such as ALD and laser etching, leveraging their atomic-level processing precision or unique structural design capabilities, are paving new ways for the design of next-generation intelligent and ultra-efficient EMI shielding materials. Compared to conventional processes, these new strategies enable more refined, controllable, and multifunctional interfacial structure regulation on fiber surfaces, thereby further expanding the application prospects of EMI shielding fibers in fields such as intelligent protection, optical stealth, and multifield response [198–201]. He et al. constructed a nacre-inspired functional layer on the CF surface using ALD technology, realizing the preparation of colored CFs while endowing them with excellent EMI SE_T_ and optical camouflage characteristics. This study demonstrated that ALD can deposit ultra-thin and uniform inorganic layers on fiber surfaces with precise thickness controllability and excellent interfacial adhesion, making it an effective pathway for achieving multifunctional fiber surfaces (Fig. 11a) [202]. Other studies have shown that aluminum-doped zinc oxide (AZO) films prepared by ALD technology have excellent conductivity (DEZ:TMA ratio = 15:1, 1.053 mΩ cm), which can enhance the interfacial adhesion of the coating while achieving electromagnetic shielding mainly through conductive loss [203]. Additionally, Chen et al. utilized laser etching to in situ induce the generation of laser-induced graphene (LIG) on the surface of BF-reinforced composites, which not only enhanced the material's EMI shielding performance but also enabled visual detection of impact damage. This research showcased the advantage of laser etching for the rapid, non-contact construction of conductive networks on inorganic fiber surfaces, opening new directions for the intelligence and multifunctionality of EMI shielding composites (Fig. 11b) [204].

Compared to emerging high-precision methods like ALD and laser etching, although dipping is not novel in technical principle, it remains a highly potential functionalization means due to the simple process, low cost, and suitability for large-area continuous processing [205, 206]. As shown in Fig. 11c, Liu et al. prepared free-standing CFFs using an MWCNT dip-coating method, significantly enhancing their EMI shielding efficiency. This method is simple to operate, scalable, and can impart excellent conductivity and EMI shielding performance to the fabric while maintaining its flexibility, demonstrating the application potential of dipping as an efficient coating strategy for functionalizing fibers [207].

Although SE is a crucial performance indicator, the actual EMI shielding materials must also meet various requirements, such as flexibility, cost-effectiveness, and environmental stability. However, these characteristics often have trade-offs with EMI shielding performance (Table 4). It is worth noting that the reported EMI SE values of IHPF-based composites in different studies are often not directly comparable due to variations in testing standards and conditions. The most commonly used testing methods include ASTM D4935-18, IEEE-STD 299, and GB/T 12190, which differ in sample size, holder geometry, and frequency range (typically 8–12 GHz, 12–18 GHz, or broader). Moreover, parameters such as sample thickness, measurement configuration (coaxial transmission line; waveguide), and surface roughness can significantly influence the measured SE by altering reflection and absorption ratios. Consequently, comparing absolute SE values across different works may lead to misleading conclusions unless these variables are carefully normalized. To improve data consistency and comparability, it is recommended that future reports clearly specify the test standard, frequency range, incident wave direction, and sample thickness, and preferably include normalized parameters such as SSE, SE/t, or SSE/t. Establishing such standardized reporting practices will facilitate fair benchmarking and accelerate the rational design of EMI shielding IHPFs.

In summary, the EMI shielding functionalization of IHPFs and their fabrics primarily relies on various surface coating preparation strategies. Different processes have their own advantages and limitations in terms of coating composition control, interfacial adhesion, process cost, and scalability. From an interface engineering perspective, the performance and durability of EMI shielding IHPFs are predominantly determined by the chemical and physical attributes of the fiber–coating interface. Chemically, the intrinsic inertness of inorganic fibers often results in insufficient bonding with metallic, carbonaceous, or polymeric coatings, leading to weak interfacial adhesion and potential delamination under mechanical or thermal stress. Physically, mismatches in thermal expansion coefficients, surface morphology, and elastic moduli between the fiber substrate and the functional layer can induce microcracking, stress concentration, and interfacial failure during service. These challenges become even more pronounced in multilayer heterogeneous architectures, where repeated thermal or moisture cycling can accelerate interfacial degradation. Therefore, the future development trend lies in combining multiple strategies, conducting effective interface engineering through surface activation, gradient coating design, and atomic-level conformal deposition (such as ALD) and other approaches, to construct multifunctional electromagnetic shielding fiber materials with high efficiency, lightweight, durability, and multifield response capabilities.

## Application for EMI Shielding Inorganic High-Performance Fibers/Fabrics

**Fig. 12:**
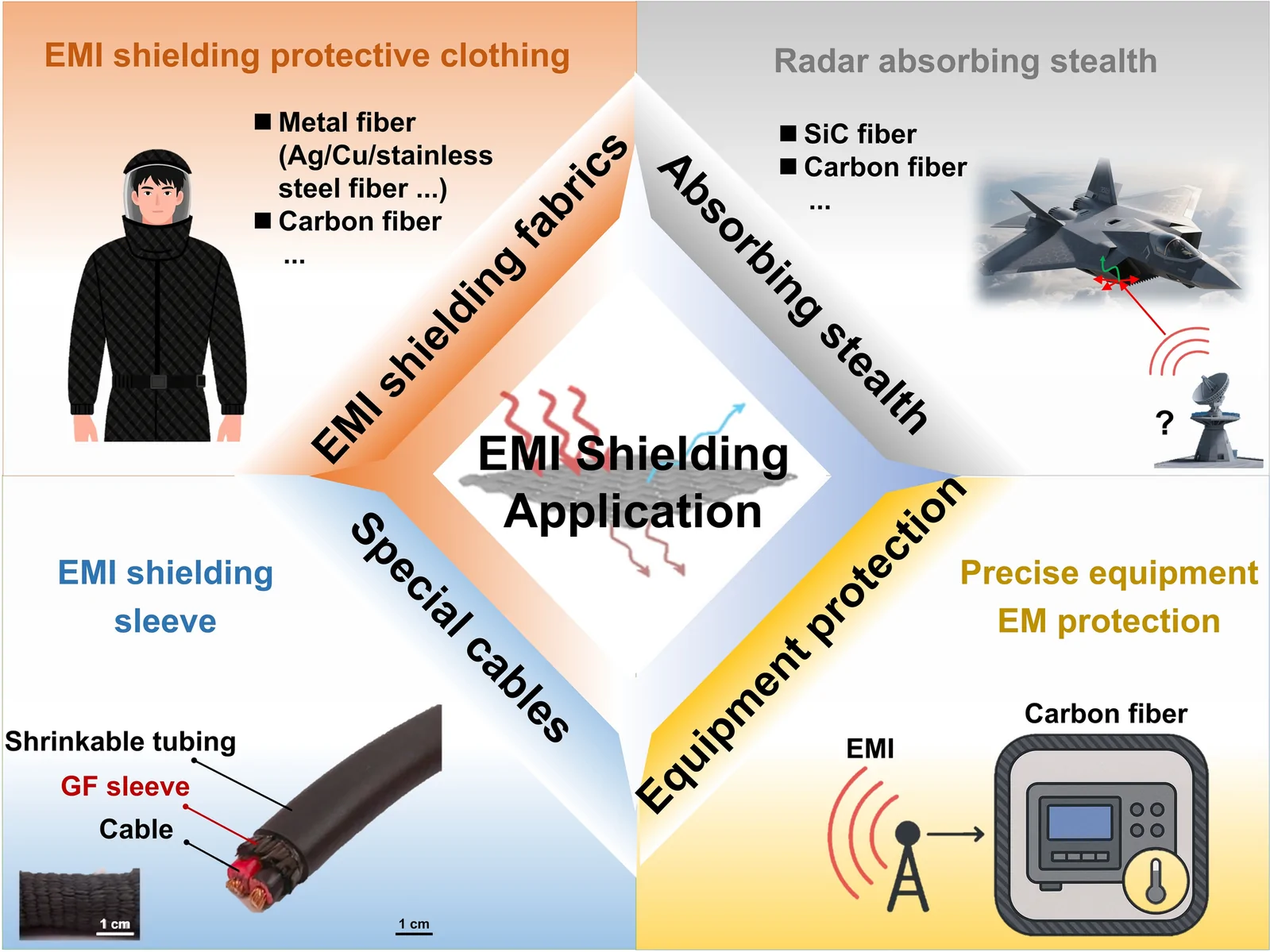
Application for EMI shielding IHPFs: EMI shielding fabrics; absorbing stealth; equipment protection; special cables

Strategies to address common challenges such as surface inertness in IHPFs are increasingly mature. Various methods for preparing functional surface coatings, including physical deposition, chemical treatments, and emerging surface modification technologies, continue to be refined. This not only provides insights for improving the interfacial bonding between fibers and functional layers but also establishes a robust processing foundation for developing composite fiber materials that integrate excellent mechanical properties with effective EMI shielding performance. Against this backdrop, EMI shielding materials based on IHPFs have gradually transitioned from laboratory exploration to practical applications, demonstrating broad prospects in several critical fields. Specifically, their applications mainly include EMI shielding textiles, wave-absorbing stealth materials, protection for precision equipment, and specialized cables (Fig. 12).

### EMI Shielding for Protection

EMI shielding textiles represent one of the most direct application avenues, widely used in products such as protective clothing, shielding curtains, and flexible shielding layers. Traditional shielding methods mostly use metals, which are not only costly, heavy, and have poor flexibility, but also not corrosion-resistant and require regular maintenance. In contrast, EMI shielding textiles based on IHPF combine structural stability with flexibility and environmental robustness. By depositing metals, conductive polymers, or magnetic materials onto fiber-based fabrics, the EM SE_T_ can be significantly enhanced while maintaining the fabric’s flexibility and breathability. CF has been widely studied and applied due to its excellent mechanical properties, conductivity, and flexibility as fabric. Mei et al. employed electroless silver plating and one-step electrodeposition techniques to develop a flexible superhydrophobic EMI shielding fabric (CEF-NF/PDA/Ag/50–30) based on polydopamine (PDA)-modified CF nonwoven fabric (CEF-NF). This fabric exhibited a SE_T_ of up to 101.27 dB in the X-band, along with a superhydrophobic surface (contact angle of 156.4°) and good breathability, making it suitable for long-term use in complex environments [208]. Fan et al. combined hydrophobic flame-retardant aramid fabric (FH-Al), porous flame-retardant finished carbonized waste cotton (CR-WCN), and carbon fiber nonwoven (CFN) using a simple sewing process to prepare a multifunctional aluminum-flammability carbonized waste cotton-carbon felt (A-FCWCF) composite fabric, which has excellent development prospects. In addition, when the A-FCWCF composite fabric’s thickness is 6 mm, the EMI SE_T_ reaches 82.63 dB, meeting the requirement of military EMI shielding materials above 75 dB [209]. In addition, BFs also demonstrate broad application prospects in the textile industry due to their mechanical properties, thermal resistance, and corrosion resistance. Xu et al. modified BFFs with functionalized anthraquinone polyurethane (WAPU) coating to produce purple BFFs with excellent wear resistance and EMI shielding function. The modified yarn did not break until 25,000 cycles, which increased the cycle life by 1462.5% compared to the original BF. The fabric maintained thermal and moisture comfort after treatment. Moreover, the WAPU/Ag coated fabric’s EMI SE_T_ reaches 41.1 dB in the X-band range [210]. Protective garments made from such materials can effectively block EMR, safeguarding personnel in specialized occupations (e.g., medical, aviation, and military fields) from EM hazards. Furthermore, owing to the excellent heat resistance, flame retardancy, and durability of IHPFs, these materials demonstrate superior overall advantages over traditional metal mesh materials in challenging environments.

In aerospace, defense, and high-end electronic equipment, precision instruments often need to operate stably in environments with strong EM pulses, radiation, and extreme temperatures. EMI shielding layers constructed from IHPFs can effectively isolate external EMI while enhancing service life through excellent thermal stability and corrosion resistance. For instance, Xiao et al. significantly improved the EMI shielding performance of SiC fiber-reinforced SiC (SiC_*f*_/SiC) composites via pyrolysis modification using inorganic salts such as Fe(NO_3_)_3_ and NH_4_Al(SO_4_)_2_. The study showed that composites with a plain-weave fabric structure modified with Fe elements exhibited optimal shielding performance in the X-band, with SE_T_ and SE_A_ reaching 39.29 and 32.36 dB, respectively. The shielding mechanism primarily involved multiple internal reflections and absorption dissipation of EM waves. In satellites, missiles, and avionics systems, multilayer shielding structures based on such modified SiC_*f*_/SiC fabrics can simultaneously achieve lightweight design, high-temperature stability, and strong EM protection. Compared to traditional metal shielding layers, their flexibility and wearability make them more suitable for covering complex structures and irregularly shaped equipment, demonstrating great potential for protecting precision devices in extreme environments.

EMI shielding cables and shielding sleeves are critical components in EM compatibility design, widely used in military communications, rail transportation, and medical electronic equipment. IHPF-based fabrics, owing to their excellent mechanical properties and abrasion resistance, are ideal skeleton materials for flexible shielding sleeves. When combined with metal or conductive coatings, they effectively reduce EM leakage and signal interference. Moreover, these materials maintain stable performance under extreme conditions, providing reliable protection for critical circuits and data transmission. Gao et al. prepared GO fibers via wet spinning and obtained a series of structurally optimized graphene fibers through chemical reduction and high-temperature annealing, significantly enhancing the electrical conductivity of the fibers. Due to the high EMI shielding performance and flexibility of monolayered textiles woven from graphene fibers, they can be wrapped around cable bundles and protected by heat-shrink tubing, showing potential for applications in daily life and aerospace signal transmission. Furthermore, the standardization and optimization of weaving technology will promote the functional advantages of graphene fiber textiles in EMI shielding, accelerating iterative development in areas such as personal protection and information security.:

### EM Wave-Absorbing Stealth

EM wave absorption and stealth technology are core requirements in aerospace and defense applications. Generally, electromagnetic shielding materials mainly suppress EMI through the reflection of conductive layers and multiple scattering, blocking the transmission of electromagnetic waves. On the other hand, electromagnetic wave absorption materials focus on converting incident electromagnetic energy into heat or other forms through dielectric and magnetic loss mechanisms, thereby minimizing secondary reflections and achieving radar stealth performance [211–218]. When combined with magnetic particles, carbon-based nanomaterials, or dielectric materials, IHPFs can form lightweight, broadband wave-absorbing structures used for EM stealth in fighter aircraft, drones, and naval vessels. Luo et al. coated magnetic Fe-Co alloy on CF’s surface (FeCo@CFs) through electroplating. By adjusting the electroplating temperature at 25, 35, and 50 °C, they obtained thin plates, irregular particles, and pyramids as Fe-Co coating morphologies, and FeCo@CFs with different coating morphologies exhibited different magnetic and complex dielectric constants. Among them, FeCo@CFs with a thin plate morphology showed the best absorption performance of 37.7 dB at 2–18 GHz [219]. Liu et al. successfully prepared CF/epoxy composites with tunable electrical conductivity by controlling the temperature and duration during the low-temperature carbonization stage of polyacrylonitrile (PAN) pre-oxidized fibers. This approach enabled a shift from traditional EM shielding to highly efficient microwave absorption. By precisely regulating the microstructure and graphitization degree of the CFs, the impedance matching performance was significantly improved, resulting in excellent wave-absorbing properties across multiple frequency bands [220]. Despite their promise as green and multifunctional EMI protection solutions, absorption-based systems also face several intrinsic limitations. Their absorption bandwidths are often restricted by impedance matching constraints and frequency-selective losses, making broadband absorption challenging under variable electromagnetic conditions. Moreover, thermal management becomes critical under high-power irradiation, as excessive heat accumulation may lead to thermal runaway, degradation of matrix resins, or magnetic loss saturation. In comparison, reflection-dominated systems, though less “stealthy,” generally offer higher stability and better heat dissipation in extreme aerospace environments due to their metallic continuity and higher thermal conductivity. Therefore, future research should focus on synergistically integrating absorption and reflection mechanisms to achieve both broadband EM attenuation and thermal robustness, fulfilling the stringent operational requirements of next-generation aerospace and defense platforms.

### Service Reliability and Environmental Stability

As everyone knows, the long-term reliability and environmental stability of EMI shielding IHPFs are critically governed by interfacial integrity and environmental degradation mechanisms. Common failure modes include delamination, oxidation, and fatigue cracking of coating layers under mechanical or thermal stress. “Wet” chemical coatings are prone to hydrolytic degradation, while metallic layers may suffer oxidation-induced conductivity loss. Strategies such as ALD barrier coatings, gradient architectures, and surface coupling agents have been shown to mitigate these effects. Systematic durability testing—such as cyclic bending, thermal shock, and salt spray exposure—is essential for establishing the correlation between microstructural stability and macroscopic shielding performance. Overall, understanding and addressing degradation mechanisms are essential foundations for the practical deployment of EMI shielding IHPFs in advanced engineering systems.

In summary, EMI shielding materials based on IHPFs show broad application prospects in flexible textiles, wave-absorbing stealth, precision equipment protection, and specialized cables. However, in practical applications, there are many challenges regarding the long-term reliability and environmental stability of EMI shielding IHPF-based composites. The interfaces among fibers, functional coatings, and matrix resins represent the most critical yet vulnerable regions, where cyclic thermal loads, mechanical vibrations, and humid environments can induce microcracking, delamination, or oxidation. Accordingly, precise chemical and physical interface engineering is indispensable to achieve large-scale practical engineering applications and maintain functionality under complex operating conditions. Techniques such as ALD for conformal nanocoating, gradient interfacial architectures for stress relaxation, and coupling-agent-assisted bonding for enhanced chemical compatibility have demonstrated significant improvements in adhesion strength and mitigation of interfacial degradation. By integrating these interfacial optimization approaches into both fabrication and application processes, IHPF-based EMI shielding materials can attain superior durability, multifunctionality, and environmental resilience—bridging the gap between laboratory demonstrations and real-world implementation in aerospace and defense systems. In the future, with continued advancements in multifunctional coating technology, IHPFs are expected to play an even more critical role in integrated systems for EM shielding, protection, and intelligent monitoring.

## Summary and Outlook

Owing to their lightweight nature, high specific strength, high modulus, and excellent environmental stability, IHPFs have become indispensable key materials in extreme environments such as aerospace and defense/military applications. They provide cutting-edge solutions for related equipment in terms of lightweight design, high durability, and multifunctionality. This review briefly introduces the mechanisms of EMI shielding, highlights common issues such as the surface inertness of IHPFs, and elaborates on both “dry” and “wet” surface modification strategies. These modification strategies enable the construction of robust functional layers on the fiber surfaces, integrating high strength, high modulus, and multifunctionality, while ensuring the interfacial reliability and stability of the composites. Subsequently, we reviewed the principles and processes of various preparation strategies for constructing EMI shielding functional layers on fiber surfaces, discussed in depth how different methods tailor the EM parameters and microstructure of the materials, and summarized the application progress of EMI shielding functionalized IHPFs in areas such as EMI shielding textiles, wave-absorbing stealth, precision equipment protection, and specialized cables.

**Fig. 13:**
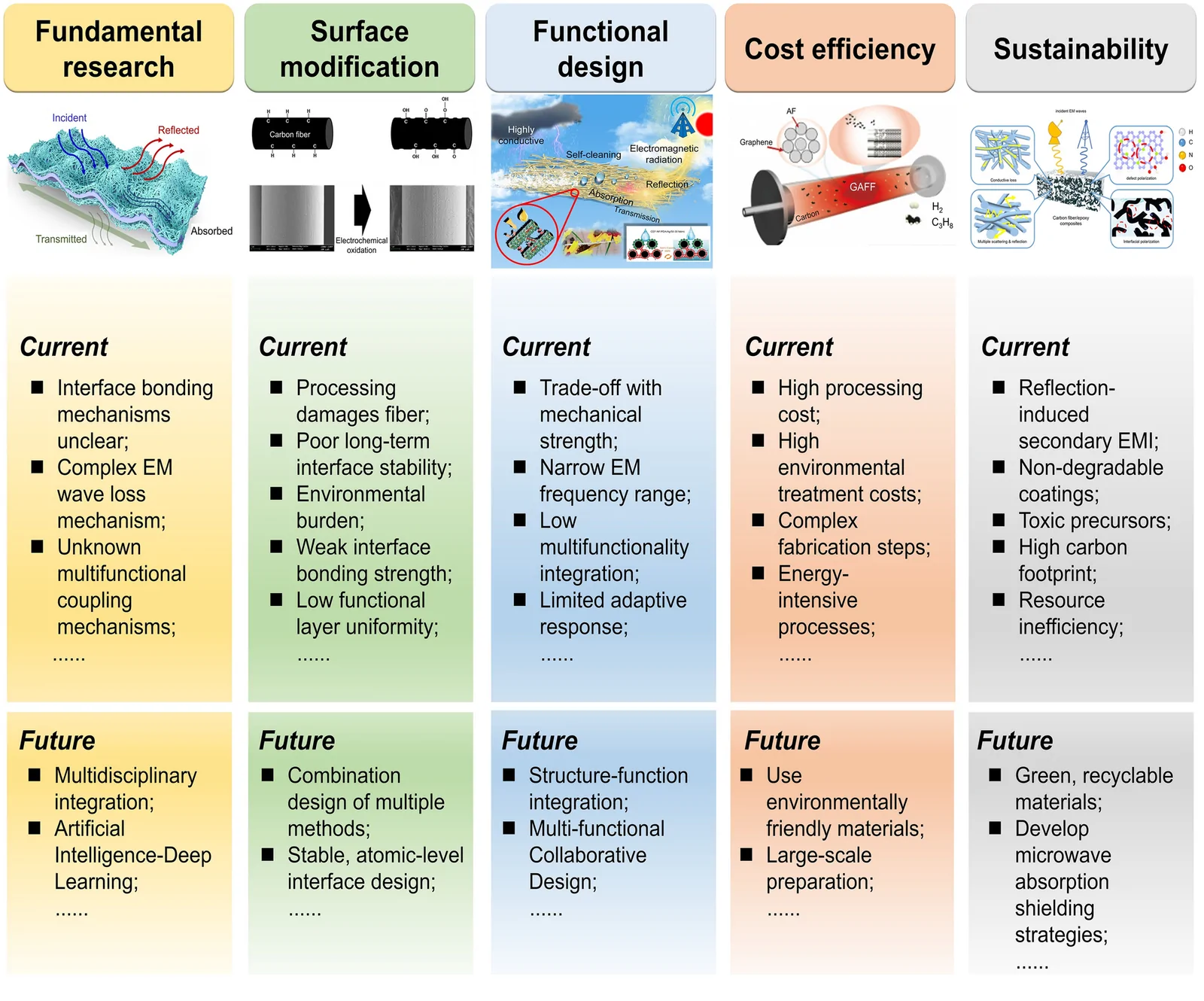
Summary of current research progress and future challenges and perspectives for EMI shielding IHPF-based materials

The development of IHPFs has achieved remarkable accomplishments over the past few decades. Their exceptional mechanical properties, favorable cost-effectiveness, and potential for multifunctional integration have gradually established them as crucial components of a new generation of advanced composite materials. With the growing severity of EMI and EMR issues, the strategic value of EMI shielding functionalization for IHPFs in extreme environments has become increasingly prominent. However, several key challenges require breakthroughs to facilitate their large-scale and long-term application in high-precision and advanced fields: (1) Further optimization of material design and structure is needed to ensure the long-term stability of the interfaces between the functional layer and the fiber, and between the fiber and the resin matrix. (2) Strategies for functional design and processing must be refined to maintain excellent mechanical properties while balancing EMI shielding and absorption performance. (3) Functional layers need to evolve beyond single shielding functions toward the integrated synergy of EMI shielding, absorption, and other multifunctional capabilities. (4) Cost control and the realization of scalable manufacturing are essential alongside the functionalization process. (5) A shift from shielding modes relying solely on EM wave reflection toward absorption-dominated green shielding modes is necessary to achieve environmental friendliness and sustainable development (Fig. 13).

Addressing these challenges requires a multidimensional strategy that simultaneously considers interfacial design, structural optimization, multifunctional coupling, and green manufacturing. Firstly, while surface modification strategies can construct functional layers on fibers, these interfaces may still face issues like delamination, aging, and performance degradation in complex service environments. Future research should focus on optimizing the multiscale interfacial structure design between the fiber-functional layer and the fiber-resin matrix. Rational design of hierarchical and hybrid IHPFs that integrate conductive, magnetic, and dielectric components at multiple scales will be essential. Tailoring fiber composition, coating uniformity, and interfacial compatibility can help balance SE, mechanical robustness, and flexibility. Data-driven modeling and machine-learning-assisted design may further accelerate the discovery of optimal structures. For instance, combining techniques like plasma activation and atomic layer deposition to introduce chemical bonds or gradient transition layers could achieve dual assurance of chemical and mechanical stability at the interface.

Secondly, regarding the balance between mechanical properties and EMI functionality, overly thick or dense functional layers often compromise the specific strength and modulus of the fibers, while focusing solely on mechanical performance makes it difficult to achieve ideal SE. Therefore, there is an urgent need to develop functionalization strategies that offer structural controllability, lightweight, and high efficiency. Constructing ultra-thin nanocoatings, layered conductive networks, or hierarchical porous structures holds promise for granting excellent EM regulation capabilities while preserving mechanical properties, thereby achieving the synergistic optimization of “lightweight-high strength-efficient shielding”.

In addition, traditional EMI shielding primarily relies on single mechanisms like reflection or absorption. In practical applications, fiber functional layers must simultaneously address multiple demands such as mechanical reinforcement, thermal protection, corrosion resistance, and optical response. The future trend involves utilizing multiscale interface regulation and heterogeneous material synergy design to integrate EMI shielding and wave absorption performance with thermal management, fire retardancy, and sensing functionalities. However, achieving such integration inevitably introduces trade-offs between conductivity, flexibility, and strength, requiring optimized microstructures and interfacial architectures to maintain balanced performance. For instance, composites of carbon-based conductive materials (CF, CNT fiber, etc.) with metal oxides or conductive polymers can achieve coupled multimechanisms and synergistic enhancement of multiple properties.

Furthermore, concerning scalable manufacturing and cost control, while laboratory-scale techniques such as electroless plating, ALD, and CVD provide precise control at the laboratory level, their cost, industrial scalability, and energy efficiency remain critical challenges. Efforts should promote the development of green, efficient, and low-energy consumption preparation processes. Employing roll-to-roll deposition techniques, atmospheric pressure ALD, or combining spraying/dipping with microwave curing processes can enhance production efficiency and reduce costs while maintaining performance. Simultaneously, standardized testing and evaluation systems suitable for industrial production should be explored to ensure the consistency and controllability of functional fiber performance in large-scale manufacturing, thereby accelerating their practical application in aerospace, defense, and civilian sectors.

Finally, environmental friendliness and sustainability considerations must guide future material and process design. Traditional wet-chemical routes often generate chemical waste, whereas vapor-phase methods (e.g., ALD or roll-to-roll CVD) are energy-intensive. Future research should focus on developing green raw materials and biodegradable/recyclable processes. Reducing chemical emissions, utilizing recyclable or bio-based components, and adopting solvent-free coating methods will minimize environmental impact. Lightweight and corrosion-resistant IHPFs also contribute to energy savings and longer service lifetimes, aligning with green manufacturing principles. Developing low-energy, continuous, and environmentally benign processes will be vital for industrial translation. Moreover, a more critical issue is that traditional EMI shielding often relies on high-reflectivity materials, which leads to the problem of secondary scattering of EM pollution. Future research emphasis should shift toward absorption-based EMI shielding strategies, such as introducing magnetic loss, dielectric loss, and multiple scattering mechanisms to enhance the effective dissipation of EM energy. Concurrently, developing green shielding fibers based on renewable resources, low-carbon processes, and recyclable materials will contribute to advancing EM protective materials toward environmental friendliness and sustainable development.

In summary, these ongoing developments highlight that IHPFs possess immense potential for EMI shielding functionalization and beyond. However, their large-scale application in extreme environments is still constrained by multiple challenges, including interfacial stability, the coupling of mechanical and EM properties, multifunctional integration, scalable manufacturing, and green sustainability. Future development trends should focus on multiscale interface regulation and structural design, utilizing advanced processes to construct efficient functional layers while balancing lightweight and mechanical performance. For multifunctional synergy, exploring the integration of EMI shielding with thermal management, fire retardancy, sensing, and other properties is essential to meet multiple demands in complex environments and achieve functional customization. At the process level, there is a need to develop green, low-cost, and continuous functionalization technologies to facilitate the transition from laboratory research to industrial application. It is foreseeable that with continuous breakthroughs in new material systems and multidimensional structural design, the EMI shielding functionalization of IHPFs will play an increasingly vital role in aerospace, national defense security, and the next generation of intelligent electronic protection fields.

## Abbreviations

EM: Electromagnetic
EMI: Electromagnetic interference
EMR: Electromagnetic radiation
IHPF: Inorganic high-performance fiber
ALD: Atomic layer deposition
CVD: Chemical vapor deposition
SE_R_: Reflection loss
SE_M_: Multiple reflection loss
SE_A_: Absorption loss
SE_T_: Total EM shielding effectiveness
VNA: Vector network analyzer
SSE: Specific shielding effectiveness
LIG: Laser-induced graphene
GF: Glass fiber
CF: Carbon fiber
BF: Basalt fiber
QF: Quartz fiber
SiC: Silicon carbide
Al_2_O_3_: Aluminum oxide
PECVD: Plasma-enhanced chemical vapor deposition
VG: Vertical graphene
CNTs: Carbon nanotubes
MWCNTs: Multiwalled carbon nanotubes
AZO: Aluminum-doped zinc oxide
TMA: Trimethylaluminum
AgNWs: Silver nanowires
ScPEG: Solid–solid phase change polyethylene glycol
IFSS: Interfacial shear strength
ILSS: Interlaminar shear strength
HMCF: High-modulus carbon fiber
LMs: Liquid metals
ANF: Aramid nanofiber
Co–Ni: Cobalt–nickel
ACBF: Aramid-carbon blended fabric
xGnPs: Exfoliated graphene nanoparticles
PPy: Polypyrrole
PANI: Polyaniline
PTh: Polythiophene
PDA: Polydopamine
RL_min_: Minimum reflection loss
VARI: Vacuum-assisted resin infusion
MOFs: Metal–organic frameworks
MXenes: Two-dimensional transition metal carbides and nitrides
VSS: Vapor-surface–solid
VS: Vapor–solid
GO: Graphene oxide
AF: Alumina fiber
g_s_: Green shielding index
RCS: Radar cross-section
PAN: Polyacrylonitrile
EAB: Effective absorption bandwidth
DEZ: Diethylzinc
CTE: Coefficient of thermal expansion
